# Computer simulation of neutral drift among limbal epithelial stem cells of mosaic mice

**DOI:** 10.1016/j.scr.2018.05.003

**Published:** 2018-07

**Authors:** John D. West, Richard L. Mort, Robert E. Hill, Steven D. Morley, J. Martin Collinson

**Affiliations:** a Centre for Integrative Physiology, University of Edinburgh Medical School, Hugh Robson Building, George Square, Edinburgh EH8 9XD, UK; b Division of Biomedical and Life Sciences, Faculty of Health and Medicine, Lancaster University, Bailrigg, Lancaster LA1 4YG, UK; c MRC Human Genetics Unit, MRC Institute of Genetics and Molecular Medicine, University of Edinburgh, Western General Hospital, Crewe Road, Edinburgh EH4 2XU, UK; d Division of Health Sciences, University of Edinburgh Medical School, Chancellor's Building, 49 Little France Crescent, Edinburgh EH16 4SB, UK; e School of Medicine, Medical Sciences and Nutrition, University of Aberdeen, Institute of Medical Sciences, Foresterhill, Aberdeen AB25 2ZD, UK

**Keywords:** Stem cell loss, Stem cell replacement, Stem cell neutral drift, Limbal epithelial stem cell, Corneal epithelium, Loss of mosaicism

## Abstract

The use of mice that are mosaic for reporter gene expression underlies many lineage-tracing studies in stem cell biology. For example, using mosaic *LacZ* reporter mice, it was shown that limbal epithelial stem cells (LESCs) around the periphery of the cornea maintain radial sectors of the corneal epithelium and that radial stripe numbers declined with age. Originally, the corneal results were interpreted as progressive, age-related loss or irreversible inactivation of some LESC clones. In this study we used computer simulations to show that these results could also be explained by stochastic replacement of LESCs by neighbouring LESCs, leading to neutral drift of LESC populations. This was shown to reduce the number of coherent clones of LESCs and hence would coarsen the mosaic pattern in the corneal epithelium without reducing the absolute number of LESCs. Simulations also showed that corrected stripe numbers declined more slowly when LESCs were grouped non-randomly and that mosaicism was rarely lost unless simulated LESC numbers were unrealistically low. Possible reasons why age-related changes differ between mosaic corneal epithelia and other systems, such as adrenal cortices and intestinal crypts, are discussed.

## Highlights

• Age-related reduction of corneal stripes in mosaic mice was simulated.
• Stem cell loss and/or stem cell replacement reduced simulated stripe numbers.
• Stem cell replacement, without reducing stem cell numbers, caused neutral drift.
• Clumping of stem cells into larger groups caused slower decline in stripe numbers.
• Replacement rarely caused loss of mosaicism unless there were few stem cells.

## Graphical abstract

**Figure:**
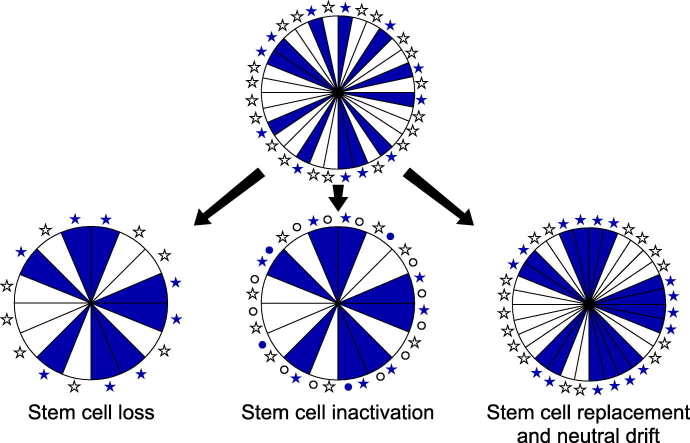

## 1 Introduction

**Fig. 1:**
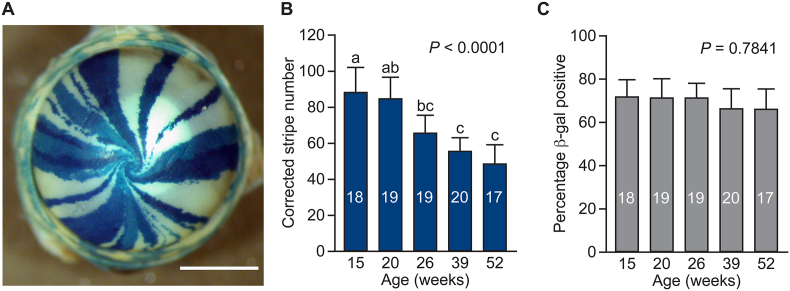
Stripe patterns in the corneal epithelium of mosaic mice. Analysis of corneal epithelial stripes in adult *XLacZ,* X-inactivation mosaic mice, modified after Mort et al. (2009) with permission of the authors and using only data from the left eyes. (A) Radial striped pattern of β-galactosidase (β-gal) staining in the corneal epithelium of an intact eye from an adult mosaic mouse. (B) Corrected stripe number in left eyes at 5 ages showing a significant reduction. (C) Percentage of β-gal positive cells in left eyes at 5 ages. Error bars are 95% confidence intervals (CI). 1-way ANOVA *P*-values are shown. Letters above the bars in (B) denote which pairs of ages differ significantly by Bonferroni post-hoc tests. Bars with only different letters (e.g. 15 vs. 26 weeks) differ significantly (*P* < 0.05) but bars with a letter in common (e.g. 15 vs. 20 weeks) do not differ significantly. Sample numbers are shown within each bar. Scale bar: 1 mm.

Two related types of observations with chimaeric or mosaic mice, or from lineage-tracing experiments, suggest that, in some tissues, progressive changes in the pattern of variegation result from stem cells being lost, irreversibly inactivated or replaced. The first observation involved the loss of one of two cell populations, from intestinal crypts of chimaeric mice (Ponder et al., 1985; Schmidt et al., 1988). This loss of mosaicism occurred between birth and adulthood and was termed ‘crypt purification’ by the authors. The second type of observation is exemplified by the age-related coarsening of variegated patterns in corneal epithelia of adult chimaeric and mosaic mice, comprising two genetically distinct cell populations (Collinson et al., 2002; Mort et al., 2009). This is as shown in Fig. 1A. Equivalent results have been reported recently, using tamoxifen-inducible lineage tracing to label K14-positive progenitor cells with the multi-coloured, R26-confetti marker at 6 weeks (Richardson et al., 2017).

Similar observations have also been made using lineage tracing to label lineages derived from zebrafish skeletal muscle stem cells (Nguyen et al., 2017) and stem cell-derived lineages in other mouse tissues (reviewed by Klein and Simons, 2011), including testis (Nakagawa et al., 2007) and intestinal epithelium (Lopez-Garcia et al., 2010; Snippert et al., 2010). In contrast, however, the stem cell-derived pattern of radial stripes in the adrenal cortex of mosaic transgenic mice did not coarsen with age (Chang et al., 2011).

Lineage tracing in intestinal crypts showed that a progressive coarsening of mosaic patterns in mixed crypts preceded loss of mosaicism, when stem cells were labelled in adults at different ages (Lopez-Garcia et al., 2010; Snippert et al., 2010). This showed that both coarsening and loss of mosaicism were time dependent rather than strictly age dependent. However, we know of no evidence that coarsening of mosaic patterns frequently leads to loss of mosaicism in other tissues and this has not been reported for the corneal epithelium (Collinson et al., 2002; Mort et al., 2009).

There is good evidence that the stem cells that replenish the mouse corneal epithelium, during normal homeostasis, reside in the basal layer of the limbal epithelium (Amitai-Lange et al., 2015; Di Girolamo et al., 2015; Dorà et al., 2015; Kasetti et al., 2016; Lobo et al., 2016; Sun et al., 2010; West et al., 2015). This is a narrow, ring-shaped transition zone between the corneal epithelium and conjunctiva, and the stem cells are known as limbal epithelial stem cells (LESCs). The pattern of radial stripes, which occurs in the corneal epithelium of mosaic mice, has been interpreted as clonal lineages of transient (or transit) amplifying cells (TACs), which are produced by LESCs at the periphery and move centripetally to maintain the tissue (Collinson et al., 2002; Mort et al., 2009).

The age-related coarsening of radial stripes in the corneal epithelium of chimaeras and mosaics was quantified by counting the number of stripes. After correction, to factor out the numbers of adjacent stripes of the same population, the corrected stripe number provides an indirect means of comparing LESC function in different groups. This is not a direct estimate of the number of active LESCs but it estimates the number of coherent clones of LESCs and is useful for comparing LESC function at different ages. The corrected stripe number in the corneal epithelium of X-inactivation mosaic mice declined with age (Fig. 1B and Mort et al., 2009). A similar decline was also demonstrated over more limited age ranges for other groups of mosaic and chimaeric mice (Collinson et al., 2002, Collinson et al., 2004; Mort et al., 2011).

This age-related decline in corrected stripe numbers in the corneal epithelium was previously interpreted as a decline in the number of active LESC clones caused either by progressive loss or irreversible inactivation of LESCs without replacement, so that each LESC maintained a larger area of the corneal epithelium (Collinson et al., 2002; Mort et al., 2009). Although these mechanisms might also account for progressive coarsening of variegated patterns reported for other tissues, such changes have mostly been attributed to stochastic neutral clonal drift without a reduction in active stem cell numbers (Klein and Simons, 2011; Lopez-Garcia et al., 2010; Nakagawa et al., 2007; Nguyen et al., 2017; Snippert et al., 2010). It has also been suggested that stochastic neutral drift might explain the reported age-related decline in corrected stripe number in the mosaic corneal epithelium (Klein and Simons, 2011; Mort et al., 2012; Richardson et al., 2017). Although this is feasible, there is currently no evidence that favours neutral drift over LESC loss, irreversible inactivation or any combination of these three mechanisms.

**Fig. 2:**
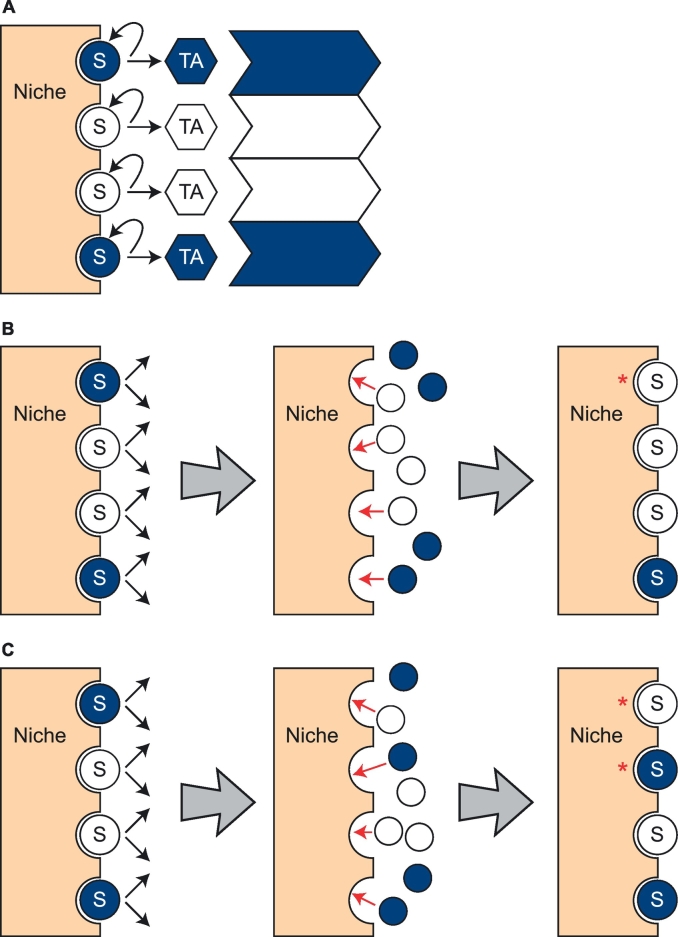
Limbal epithelial stem cell replacement may alter the mosaic corneal pattern. Diagram showing LESCs, represented by blue and white circles marked ‘S’, in a mosaic corneal limbus. Each LESC contacts the niche and transient (or transit) amplifying cells (TACs) are shown as blue and white hexagons, marked ‘TA’. The TACs produce clones of cells that extend into the corneal epithelium and form radial stripes. (A) If LESCs remain in the same position and always divide asymmetrically (to produce one LESC and one TAC) the corneal stripe number should not change with age, unless LESCs are inactivated or die and are not replaced. In the example shown, 2 blue and 2 white LESCs form 3 LESC patches and 3 corneal stripes (blue and white arrows). (B,C) In the alternative hypothetical possibilities illustrated, LESCs produce two daughter cells that are initially equivalent and compete to occupy limited space in the niche. Those that secure contact with the niche (red arrows) become LESCs and others become TACs. This would sometimes result in stochastic replacement of an LESC by a daughter cell of a neighbouring LESC (red asterisks). Three sequential steps are shown from left to right. Any changes in the LESC patch number will subsequently affect the uncorrected corneal stripe number. In (B) the top blue LESC is replaced by the adjacent white LESC, so the percentage of blue LESCs and the LESC patch number decrease. In (C) the top two LESCs exchange places so the pair of white LESCs is split. The percentage of blue LESCs remains unchanged but the LESC patch number increases. (B) and (C) are simplified illustrations of a hypothetical mechanism. Although biological LESC divisions are unlikely to be completely synchronous, the type of LESC replacements illustrated could occur if some LESC divisions result in a niche position remaining empty until the neighbouring LESC divides. The simulation model we describe neither assumes nor requires synchronous division of LESCs. The figure is modified after Mort et al. (2012). (For interpretation of the references to colour in this figure legend, the reader is referred to the web version of this article.)

Stochastic neutral drift could occur in the corneal limbus if some LESCs were replaced by neighbouring LESC lineages without any net loss in LESC numbers. This might usually require some LESCs to divide symmetrically, to produce two LESCs or two TACs, rather than asymmetrically, to produce one LESC and one TAC. Population asymmetry would be maintained if the two types of symmetric LESC divisions were balanced and this could be regulated either cell-autonomously or by extrinsic factors, as discussed by Klein and Simons (2011). One hypothetical type of extrinsic regulation is illustrated in Fig. 2, to show how LESC replacement might occur.

However, it is unclear whether LESC replacement would affect the quantitative changes in corrected stripe number in the corneal epithelium in the same way as the uncorrected stripe number. This is because LESC replacement is likely to change the proportions of the two LESC populations and this would affect the correction factor and so would alter the relationship between the uncorrected and corrected stripe numbers. Furthermore, as LESC replacement might increase, as well as decrease, the number of stripes in a mosaic corneal epithelium (Fig. 2), it is not intuitively obvious that it would inevitably result in loss of mosaicism, even after a large number of LESC generations.

Our main aim was to test the hypothesis that stochastic LESC replacement, leading to neutral drift, could account for the observed reduction in corrected stripe numbers in the corneal epithelium. For this, we simulated the effects of stochastic LESC loss (without replacement) and stochastic LESC replacement in a simulated mosaic limbal epithelium. This comprised two simulated LESC populations that were distributed around a limbal ring, either randomly or in coherent clonal groups. We then determined the consequences for the proportions of the two LESC populations, the uncorrected stripe number and the corrected stripe number. Our secondary aim was to consider why ageing affected mosaic patterns in the mouse adrenal cortex, corneal epithelium and intestinal crypts differently. For this, we investigated variables that slowed the reduction in corrected stripe numbers or favoured loss of mosaicism in the simulations.

## 2 Materials and methods

### 2.1 Assumptions of the computer model

Some β-galactosidase (β-gal) positive stripes in mosaic corneas appear paler than others (Fig. 1A) and this has been attributed to clonal variation in transgene expression (Mort et al., 2009). However, only two populations of stem cells (blue and white) were simulated. This is consistent with biological studies with this mosaic system, which did not distinguish between β-gal-positive cells with different levels of staining (Collinson et al., 2002; Mort et al., 2009).

To mimic our previous studies, the computer model simulates the distribution of two LESC populations (‘blue’ and ‘white’) that occupy the basal layer of the narrow ring of limbal epithelium, in a *LacZ* mosaic mouse. In a mouse, the LESCs would produce blue and white TACs, which would move into the corneal epithelium and across the radius to the centre. The mouse corneal epithelium is about 5–6 cells thick but the striped patterns in corneas of mosaic mice (Fig. 1A) are effectively 2-dimensional, because the upper more differentiated layers are derived directly from the underlying basal layer of TACs. Two-dimensional patterns of radial stripes can be represented by 1-dimensional rings so, for example, the ring at the border between the limbus and the corneal epithelium represents the distribution of early generation TACs, where they enter the corneal epithelium. In the mouse, the basal layer of limbal epithelium between the corneal epithelium and conjunctiva forms a narrow 2-dimensional annulus rather than a 1-dimensional ring. However, for the purposes of the simulation, we assume that the LESCs form a 1-dimensional ring and each LESC has only two neighbouring LESCs.

In the simulated 1-dimensional ring of blue and white LESCs, a ‘patch’ of LESCs is defined as an uninterrupted sequence of contiguous LESCs of the same population (blue or white) around the circumference. We assume that each blue or white patch of simulated LESCs would produce an equivalent blue or white stripe in the corneal epithelium, so the LESC patches and corneal stripes are numerically equivalent. Although the model simulates the distribution of LESC patches, for consistency with terminology used for studies of mosaic mice (Fig. 1), we refer to the equivalent number of corneal stripes when describing the simulation outputs. To show how the computer output would be interpreted as equivalent corneal epithelial stripes, the effects of stochastic LESC replacement, on a small array of LESCs and the resultant corneal epithelial stripe pattern, are illustrated in Fig. S1.

The stripes will tend to be coarser if LESCs are grouped into coherent clones, comprising multiple LESCs of the same population, and also if stripes contain multiple adjacent coherent LESC clones of the same population. Mosaic corneas, with LESCs grouped into multicellular coherent clones, were simulated in ‘clumped arrays’, as described below. In biological studies of labelled/unlabelled mosaics, the observed stripe number was corrected to factor out effects of random adjacency of multiple coherent clones of the same population, using the correction factor 1/(1-p), where p is the proportion of labelled cells around the circumference and 1/(1-p) is the predicted mean number of adjacent, labelled coherent clones (Collinson et al., 2002; Mort et al., 2009). The same approach was used to calculate the ‘corrected stripe number’ in the computer model.

### 2.2 Computer simulation

The web app ‘CloneSim’ was written in JavaScript with Angular JS and was designed to run on the web browser, Google Chrome. For each simulation, a 1-dimensional circular array (closed linear array) was established to represent a mixture of two populations of LESCs (referred to as ‘blue’ and ‘white’ or ‘positive’ and ‘negative’ stem cells) at G0. The following parameters were varied at set-up: (1) the number of LESCs in the array at generation 0 (G0); (2) the number of subsequent LESC generations (division iterations) to be simulated; (3) the initial proportion of positive (blue) LESCs; (4) the number of LESCs per coherent clone (the value is set at 1, to simulate random distributions, or higher, to simulate coherent clonal groups); (5) the probability of an LESC being replaced by a neighbouring LESC and (6) the probability of an LESC being lost (and not replaced).

A defined probability of stochastic LESC loss (without replacement) and/or stochastic LESC replacement (whereby an LESC was replaced by one that was equivalent to an adjacent LESC) was simulated at successive LESC generations without altering the initial set-up parameters. This caused changes in the array composition. Simulation of stochastic LESC replacement made no assumptions about the mechanism and, for example, was not specifically designed to test the hypothetical mechanism shown in Fig. 2.

**Fig. 3:**
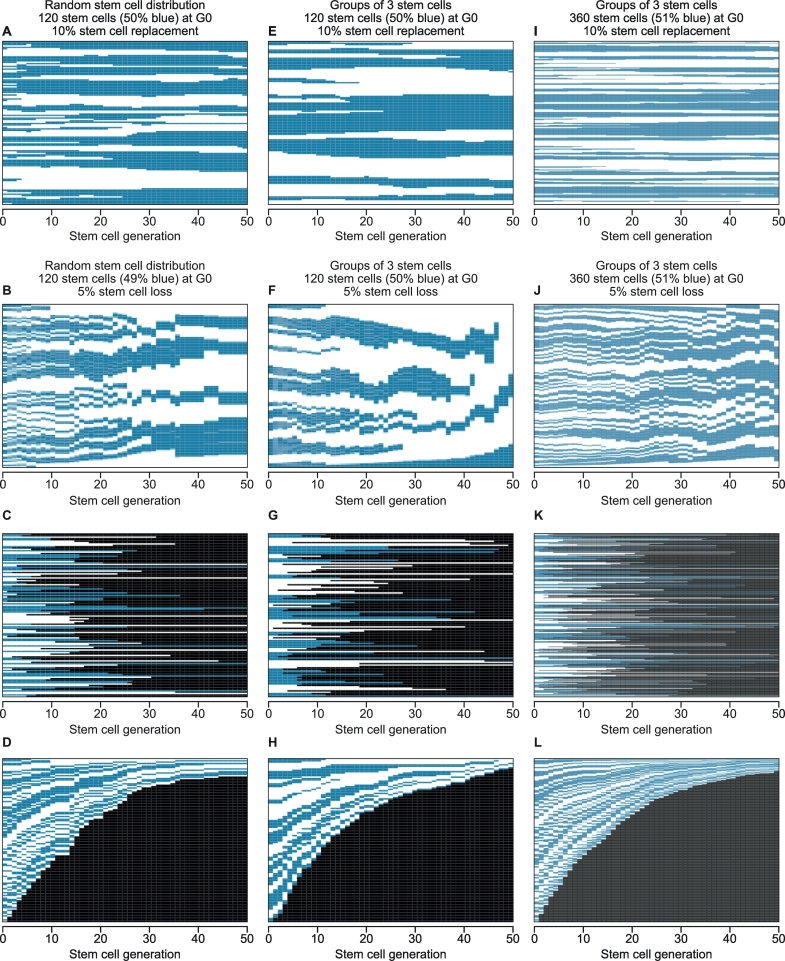
Output displays for simulations of limbal epithelial stem cell replacement and loss. Output displays for simulated changes over 50 LESC generations (on the horizontal axis) for arrays with approximately equal numbers of blue and white LESCs (49–51% blue) at generation 0 (G0). The vertical axis shows the distribution of blue and white LESCs at each generation after LESC loss or replacement. The initial stem cell distributions in the simulated arrays were (A-D) 120 randomly distributed LESCs, (E-H) 120 LESCs arranged in groups of three in ‘clumped arrays’ (random distributions of groups of three same-coloured LESCs) or (I-L) 360 LESCs arranged in groups of three in clumped arrays. (A, E, I) LESC replacement. Results for simulations with a 10% probability that any LESC would be replaced by a daughter cell of a neighbouring LESC at each of 50 LESC generations. Larger patches of the same LESC population (blue or white) are formed (vertical axis) at later generations (horizontal axis). (B-D, F-H & J-L) LESC loss. Results for simulations with a 5% probability that any LESC would be lost (and not replaced) at each of 50 generations. This is illustrated in three ways. In (B), (F) and (J), lost LESCs are excluded from the array so the heights of the remaining blue and white LESCs are expanded to fill the vertical space. In (C), (G) and (K), lost LESCs are shown as black void spaces in line with the original LESCs. In (D), (H) and (L), lost LESCs are grouped as a single black void space at the bottom of the array to show the progressive loss of LESCs over 50 LESC generations more clearly. (For interpretation of the references to colour in this figure legend, the reader is referred to the web version of this article.)

The software displayed the distribution of LESCs (on the vertical axis) as blue and white rectangles for each simulated LESC generation (shown on the horizontal axis). Numerical data, showing the LESC population as a binary code (1 for blue or 0 for white), for each LESC position in the array at each generation, plus 12 summary parameters (listed in Table S1) were downloaded as CSV files. Images of the displays were downloaded as PNG files and examples are shown in Fig. 3.

For each LESC generation, the summary output included the proportion of blue LESCs in the array, the uncorrected stripe number (blue plus white stripes) and the corrected stripe number, which was calculated as described previously (Collinson et al., 2002; Mort et al., 2009). The quantitative effect of correcting the stripe numbers, generated by the simulations, is illustrated in Fig. S2 for simulated random arrays, at generation (G0), without any LESC loss or replacement. Unlike the uncorrected stripe number, the corrected stripe number is independent of the percentage of blue cells in the array, so is more suitable for comparing different groups. For random distributions of blue and white LESCs, there is only one LESC per coherent clone so the estimated corrected stripe number should equal the total number of simulated LESCs in the array at G0, provided the array is large enough and the distribution is truly random.

### 2.3 Analysis of computer simulation output

The total number of blue plus white LESCs in the array remained unchanged in simulations of LESC replacement but declined in simulations of LESC loss. For generations where one LESC population was lost, the percentage of blue LESCs was recorded as 0 or 100, the uncorrected stripe number was recorded as 1, rather than 0, and the corrected stripe number was not calculated. For generations where both LESC populations were lost, the percentage of blue cells, the uncorrected stripe number and the corrected stripe number were not calculated so they were not included in the group means. Twenty repeat simulations were used to evaluate each combination of variables and only arrays with the correct target percentage of blue cells were used. Where the same combination of variables was included in more than one series of comparisons, a separate set of 20 simulations was run for each series.

The half-life of the corrected stripe number, expressed in LESC generations, was defined as the first LESC generation where the corrected stripe number was half or less than half of that at G0. In most cases the half-life was determined for the mean corrected stripe number for a set of 20 simulations and is shown as t_1/2_. In some cases, the half-life was determined separately for each simulation and is shown as the t_1/2_(i) for an individual simulation or the mean t_1/2_(i) for a set of simulations. Half-lives, for the LESC number and the uncorrected stripe number, were derived in an equivalent way.

### 2.4 Choice of parameters for the computer model

To better quantify the biological decline in corneal epithelial corrected stripe numbers, results shown in Fig. 1B were re-plotted on a linear axis from five weeks (Fig. S3), when LESCs are likely to be activated (Collinson et al., 2002). This suggested a decline from a corrected stripe number of 124 at five weeks to half this number by about 31 weeks, implying that the corrected stripe half-life (t_1/2_) is about 26 weeks. It is difficult to convert this half-life into LESC generations, as the frequency of LESC divisions is unknown. One estimate can be derived by assuming that LESCs are equivalent to label-retaining cells, in the mouse corneal limbus, in which BrdU remains detectable by immunohistochemistry, after a 10-week chase period (Douvaras et al., 2013). If it is assumed that BrdU is diluted to levels that are undetectable by immunohistochemistry after 4–5 cell divisions, as reported for FACS (Wilson et al., 2008), label retention for 10 weeks implies that, at least, some LESCs do not divide more often than once per two weeks. On this basis, the corrected stripe number half-life of 26 weeks would be equivalent to no more than 13 LESC generations and about 50 LESC generations would occur during the typical two-year maximum lifespan of a laboratory mouse.

Most label-retaining cells are expected to be slowly dividing or relatively quiescent LESCs. However, some tissues, such as the intestinal epithelium, have stem cells that divide quickly (Barker et al., 2008). More recently, it has become apparent that tissues may have both slowly and more rapidly dividing stem cells (Krieger and Simons, 2015). If this is true of the corneal limbus, the label-retaining cells may identify slow cycling but not faster cycling stem cells. In addition, LESCs may alternate between active and quiescent states (Dorà et al., 2015). In this case, label-retaining cells will identify those that were cycling during the labelling period and then became quiescent for several weeks, regardless of whether they cycled quickly or slowly during their active state. If some LESCs divided more rapidly than estimated from the label-retaining cell experiments, more than 13 LESC generations will occur during the 26-week corrected stripe number half-life. We, therefore, simulated a range of corrected stripe number half-lives.

The number of individual stripes, which could fit around the mouse corneal epithelium, has been estimated from mouse lineage-tracing experiments as either approximately 100 (Amitai-Lange et al., 2015) or 250–300 (Dorà et al., 2015). As the higher estimate is about three times that of the corrected stripe number in corneas from mosaic mice of a similar age (Collinson et al., 2002; Mort et al., 2009), LESCs could be clustered in coherent clonal groups with an average of about three LESCs per coherent clone. We, therefore, included simulations of LESCs clustered into groups (clumped arrays) as well as simulations of LESCs that were randomly distributed.

### 2.5 Statistical analysis

The choice of parametric or non-parametric tests was guided, in part, by normality tests. GraphPad Prism version 5.0c (GraphPad Software Inc., La Jolla, USA) was used for most statistical tests, including 1-way analysis of variance (ANOVA), 2-way ANOVA, Kruskal-Wallis test, 2 × 2 Fisher's exact test and chi square test for trend. The log-rank (Mantel-Cox) test and log-rank test for trend were used for survival distributions. An online statistical calculator (Lowry, 2017) was used for Fisher's exact test with tables larger than 2 × 2. The error bars in the figures are 95% confidence intervals (CI) unless stated otherwise.

## 3 Results

### 3.1 Simulations of LESC loss or LESC replacement caused a decline in stripe numbers

**Fig. 4:**
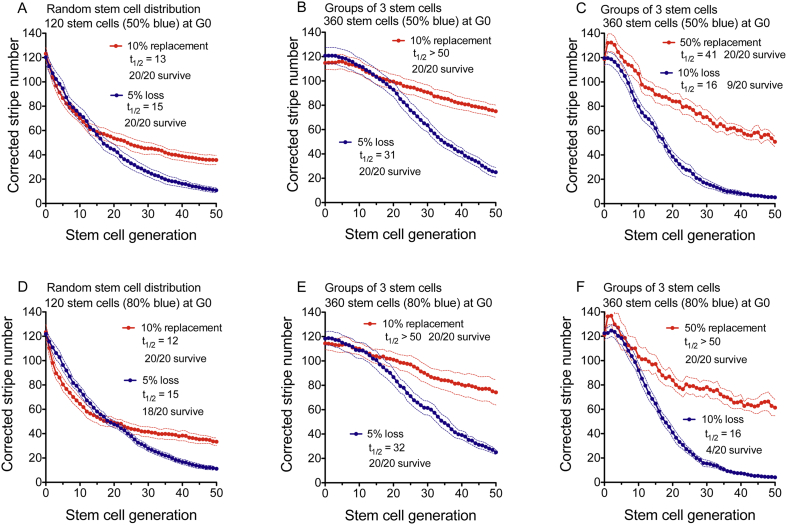
Decline in corrected stripe number in simulations of limbal epithelial stem cell loss or replacement. The effects of LESC loss or replacement on the corrected stripe number (mean ± 95% CI) are shown over 50 LESC generations for 12 sets of 20 simulations for arrays initiated with 50% blue LESCs (A-C) or 80% blue LESCs (D-F). (A,D) 5% LESC loss or 10% LESC replacement in simulations of 120 LESCs that were randomly distributed at generation 0 (G0). (B,E) 5% LESC loss or 10% LESC replacement in simulations of 360 LESCs in clumped arrays of groups of three (random distributions of groups of three same-coloured LESCs, rather than random distributions of single LESCs) at G0. (C,F) 10% LESC loss or 50% LESC replacement in simulations of 360 LESCs in clumped arrays of groups of three LESCs at G0. Corrected stripe number half-lives are shown as t_1/2_ LESC generations and were determined for the mean corrected stripe number of the set of 20 simulations as described in the Materials and Methods. The frequency of simulations where both cell populations survived for 50 LESC generations is shown after the half-life. As the corrected stripe number is not calculated if either LESC population is lost (see Materials and Methods), the mean corrected stripe number is based on <20 simulations in later cell generations of the cell loss simulations shown in (C), (D) and (F). (For interpretation of the references to colour in this figure legend, the reader is referred to the web version of this article.)

Arrays of 120 randomly distributed blue and white LESCs were simulated, to approximate the predicted initial corrected stripe number of 124 (Fig. S3). Larger array sizes were used for simulations where LESCs were in ‘clumped arrays’ to maintain a similar LESC coherent clone number and, therefore, a similar predicted corrected stripe number in the corneal epithelium. We first compared the effects of LESC loss and LESC replacement on the corrected stripe number, over 50 LESC generations, for random arrays and clumped arrays with groups of three LESCs (Fig. 4). Both balanced arrays, with 50% blue LESCs at G0 (Fig. 4A–C), and unbalanced arrays, with 80% blue LESCs (Fig. 4D–F) were included. In simulations of random arrays, a 5% probability of LESC loss (per LESC per generation) or a 10% probability of LESC replacement (per LESC per generation), initially reduced the corrected stripe number at similar rates with half-lives of 12–15 LESC generations (Fig. 4A,D). However, at later LESC generations, the decline in corrected stripe number was slower for 10% LESC replacement. Comparable simulations, but with higher corrected stripe number half-lives, were produced by reducing the probability of LESC loss or replacement (Fig. S4). These simulations showed that either stochastic LESC loss or stochastic LESC replacement, leading to neutral drift, could account for the observed reduction in corrected stripe numbers in the corneal epithelium.

### 3.2 Clumping of LESCs into larger groups caused a slower decline in stripe numbers

In clumped arrays, with groups of three LESCs (Fig. 4B,E), the decline in corrected stripe number was slower than in equivalent random arrays (Fig. 4A,D) and it was often preceded by a short period when the corrected stripe number remained relatively stable or even increased slightly (Fig. 4B,C,E,F). The corrected stripe number half-life was similar in clumped arrays with 10% LESC loss (Fig. 4C,F) to random arrays with 5% LESC loss (Fig. 4A,D) as the initial lag was followed by a steeper decline. The slower decline in clumped arrays was more pronounced for LESC replacement than LESC loss and, even with 50% LESC replacement in clumped arrays (Fig. 4C,F), the initial decline remained slower than in random arrays with 10% replacement (Fig. 4A,D). For unbalanced arrays with 80% blue cells (Fig. 4D–F), the effects of LESC loss and LESC replacement on corrected stripe numbers were similar to those for the balanced arrays (Fig. 4A–C).

### 3.3 Further analysis of simulated LESC loss and LESC replacement

More detailed analyses of changes that occurred during the simulations of LESC loss and LESC replacement, shown in Fig. 4, are provided in the supplementary information (Figs. S5–S9). The decline in uncorrected stripe numbers was similar to that for corrected stripe numbers in simulations of balanced and unbalanced arrays, (Figs. S5 and S6). Similar to the biological results (Fig. 1C), the mean percentage of blue cells was not affected greatly by LESC replacement but became more variable when cell numbers were reduced in simulations of LESC loss (Figs. S5 and S6). Both LESC populations survived in all the simulations of LESC replacement shown in Fig. 4 but one or both LESC populations were lost in some of the simulations of LESC loss (Fig. S7). For cell loss in random arrays of 120 LESCs, corrected stripe numbers (Fig. 4A,D) and uncorrected stripe numbers (Fig. S5A, S6A) declined with similar half-lives to LESC numbers (Fig. S7A).

In individual simulations, LESC loss almost always caused the uncorrected stripe number to remain unchanged or decline by a multiple of two at each generation (Fig. S8A–F). The only exceptions were in LESC generations where one LESC population was lost, so the uncorrected stripe number declined from two to one. In contrast, LESC replacement could increase as well as decrease the uncorrected stripe number in individual simulations at some cell generations (Fig. S8G–L). In clumped arrays, where all stripes are initially at least three LESCs wide, uncorrected stripe numbers did not decline between G0 and G1 (Fig. S8), which explains the early lag in mean uncorrected stripe number (Fig. S5D,G). Individual changes in corrected stripe numbers were more variable than changes in uncorrected stripe numbers and both simulations of LESC loss and LESC replacement caused some individual increases as well as decreases (Fig. S9). This additional variation is presumably because the corrected stripe number need not be an integer and is a calculated value, with associated error.

### 3.4 Further analysis of the effects of LESC clumping

**Fig. 5:**
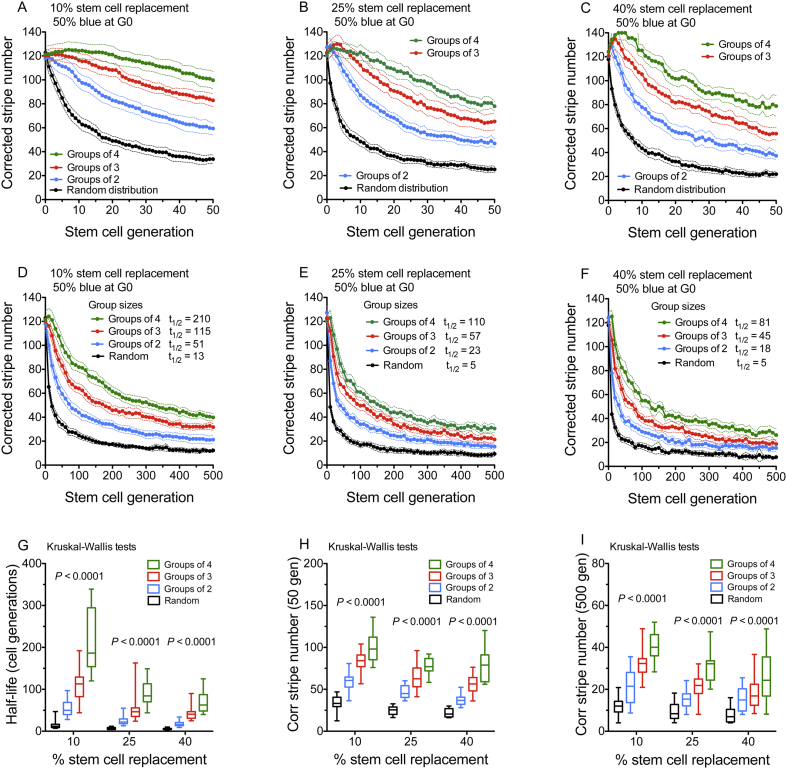
Effect of initial limbal epithelial stem cell distribution on the decline in corrected stripe number in simulations of LESC replacement in arrays with 50% blue cells at G0. (A-F) Comparison of decline in corrected stripe numbers (mean ± 95% CI; 20 simulations per set) over 50 (A-C) and 500 (D-F) LESC generations for four different types of array distributions with 50% blue cells at G0. Random arrays had 120 LESCs and clumped arrays with groups of 2, 3 or 4 cells had 240, 360 and 480 LESCs respectively. These array types were compared for simulations of 10% (A,D), 25% (B,E) and 40% (C,F) LESC replacement. In (D-F), data were plotted for every tenth generation. Both LESC populations survived for 500 cell generations in all simulations. (G-I) The corrected stripe number half-life (G), corrected stripe number at 50 LESC generations (H) and corrected stripe number at 500 LESC generations (I) were compared among the four different array distributions. The non-parametric Kruskal-Wallis test was used because some results were not normally distributed (*P*-values are shown). Half-lives shown as t_1/2_ LESC generations in (D-F) were defined as explained in the Materials and Methods and determined for the mean of the set of 20 simulations but half-lives in (G) were determined separately for each simulation. Box and whisker plots in (G-I) show the median (horizontal line within the box), upper and lower quartiles (top and bottom of boxes) and the minimum and maximum of all the data (ends of whiskers). (For interpretation of the references to colour in this figure legend, the reader is referred to the web version of this article.)

The moderating effect of LESC clumping on the decline in corrected stripe numbers caused by LESC replacement, shown in Fig. 4, was investigated further by comparing random arrays and clumped arrays, with groups of 2, 3 or 4 LESCs (to simulate different coherent clone sizes), over 500 LESC generations. For each of three LESC replacement probabilities (10%, 25% and 40%) in balanced arrays, with 50% blue cells at G0, there was an inverse relationship between LESC group size and the initial rate of decline in mean corrected stripe number (Fig. 5A–F). This was reflected by significant differences, among group sizes, for the corrected stripe number half-life (Fig. 5G) and the corrected stripe number at G50 and G500 (Fig. 5H,I). The effect of array type on the decline in the mean corrected stripe number in unbalanced arrays with 80% blue LESCs at G0 (Fig. S10) was comparable to that in balanced arrays. One difference between the balanced and unbalanced arrays was that both LESC populations survived for all 500 LESC generations in each of the 12 sets of 20 simulations of balanced arrays (Fig. 5) but some of the unbalanced arrays lost the minor cell population (Tables S2, S3). This loss of mosaicism was more frequent when the probability of LESC replacement was higher but less frequent when LESCs were clustered into larger groups (Table S2).

### 3.5 Other variables that affect the corrected stripe number

Investigations of other variables that affect the decline of the corrected stripe numbers are shown in the supplementary information (Figs. S11-S18; Tables S4-S7).

First, we showed that, for clumped arrays with groups of 3 LESCs, the overall decline in corrected stripe number was similar for arrays with a wider range of percentages (10%–90%) of blue LESCs (Figs. S11 and S12). This confirmed that arrays with 50% and 80% blue LESCs, shown in Fig. 4, Fig. 5 and S5-S10, were sufficiently representative. Although the proportion of the two simulated LESC populations had no major effect on the decline in corrected stripe number, LESC replacement caused more variation in the corrected stripe number in the most unbalanced arrays (Fig. S11). Combinations of 5% LESC replacement and 2.5% loss caused rates of decline that were faster than 10% replacement but slower than 5% loss (Fig. S11).

Increasing the probability of LESC loss from 0.1% to 20% caused the expected increase in the rate of decline of LESC numbers in arrays of 120 or 360 LESCs, with a corresponding reduction in the LESC number half-life (Fig. S13A–D; Table S4). In the random arrays of 120 LESCs, corrected stripe numbers declined in parallel with the LESC numbers but the decline was slower when LESCs were clumped in groups of three in arrays of 360 LESCs (Fig. S13E–H; Table S5).

We next varied the probability of LESC replacement from 1% to 100%. For simulations with arrays of 120 randomly distributed LESCs, 30–70% LESC replacement produced the fastest and largest declines in the corrected stripe number (Fig. S14A,B; Table S6). Above and below this range the decline in corrected stripe number was slower but it was more variable for 70%–99%. For 100% LESC replacement, the corrected stripe numbers increased, rather than decreased, over 500 LESC generations. For probabilities of LESC replacement above 50%, fine-grained patterns of alternating blue and white LESCs arose (Fig. S15), which would increase the uncorrected and corrected stripe numbers over this range.

In clumped arrays, the trends were similar but the declines in corrected stripe number were slower. Again, probabilities of 30–70% LESC replacement caused the fastest declines and, for 100% LESC replacement, the corrected stripe numbers increased over 500 LESC generations (Fig. S14C,D; Table S7). There was an initial lag or increase before the corrected stripe number declined and, strikingly, the initial increase was greater and lasted for more LESC generations for higher probabilities of LESC replacement.

To investigate the effects of array size, on the rate of decline in corrected stripe number, independently of array type (random or clumped), we compared arrays of 120, 240 and 360 LESCs. These comparisons were made for simulations of random arrays and clumped arrays with groups of 2 or 3 LESCs. For simulations of 5% LESC loss (Fig. S16), 10% LESC replacement (Fig. S17), and 90% LESC replacement (Fig. S18), the corrected stripe number half-life was either unaffected or only slightly affected by the array size over this range, whereas it was much more significantly affected by array type. For simulations of LESC replacement, the corrected stripe number half-life tended to be more variable in the smaller arrays and the clumped arrays, where the corrected stripe number declined more slowly (Figs. S17G and S18G).

### 3.6 Stem cell replacement resulted in loss of mosaicism in small arrays of stem cells

**Fig. 6:**
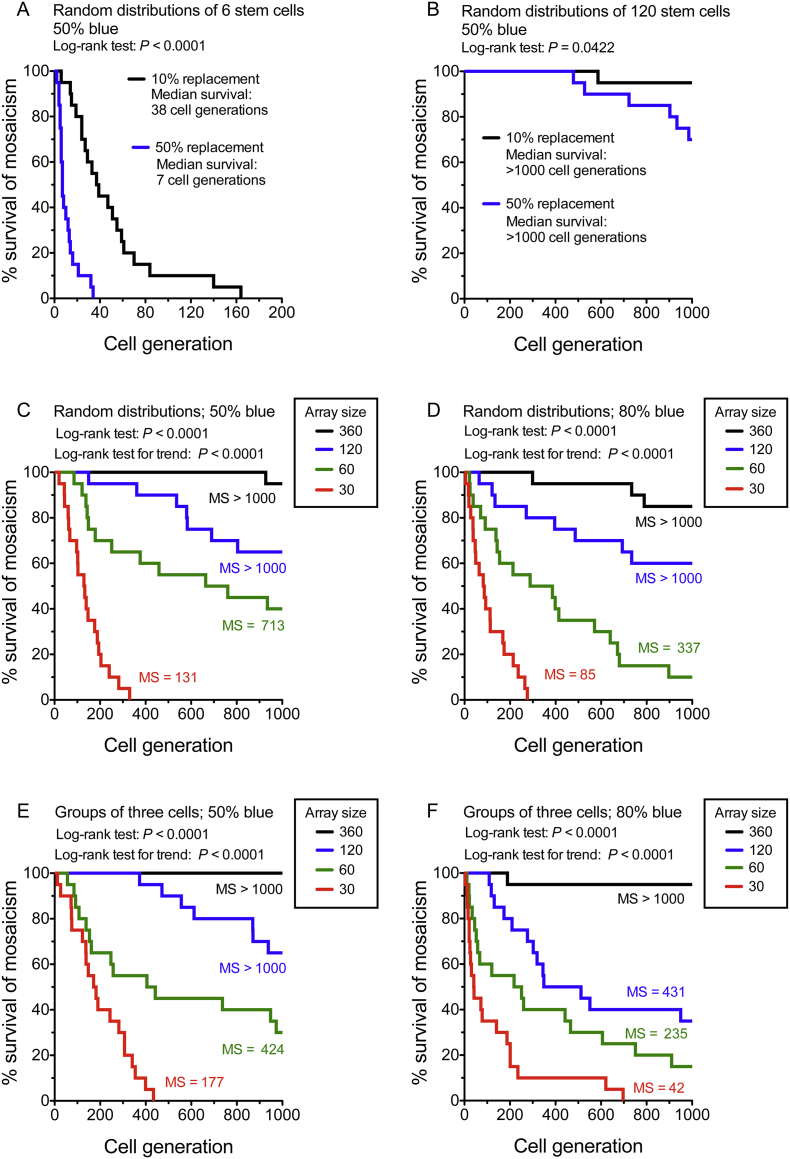
Frequency of loss of mosaicism. (A,B) Survival plots showing survival of mosaicism in 4 sets of 20 simulations with 10% or 50% probability of stem cell replacement per generation for arrays of 6 stem cells, to simulate intestinal crypt stem cells (A), or 120 cells, to simulate LESCs (B). Significance of differences between 10% and 50% stem cell replacement by the log-rank (Mantel-Cox) test are shown as *P*-values. (C-F) Survival of mosaicism in 16 sets of 20 simulations with 50% probability of stem cell replacement per generation over 1000 stem cell generations. For both random arrays (C,D) and clumped arrays with groups of 3 stem cells (E,F), with either 50% blue stem cells (C,E) or 80% blue stem cells (D,F), differences among the four array sizes were highly significant by the log-rank (Mantel-Cox) test (*P*-values are shown). Mosaicism was lost more frequently and more quickly when the array size (number of stem cells per array) was small and this trend was highly significant by a log-rank test for trend (*P*-values are shown). Abbreviation: MS, median survival of mosaicism (in stem cell generations). (For interpretation of the references to colour in this figure legend, the reader is referred to the web version of this article.)

Although mosaic patterns show a progressive coarsening in both the corneal epithelium and intestinal crypts of mosaic mice, usually this only progresses to a loss of mosaicism in intestinal crypts (Collinson et al., 2002; Mort et al., 2009; Ponder et al., 1985; Schmidt et al., 1988). It has been reported that each intestinal crypt has only 5–7 active stem cells (Kozar et al., 2013) and loss of mosaicism is attributed to stem cell replacement, leading to stochastic neutral drift (Lopez-Garcia et al., 2010; Snippert et al., 2010). To test whether the type of stem cell replacement that we simulated for LESCs could account for loss of mosaicism in a small 1-dimensional ring of stem cells we compared the frequency of loss of mosaicism in simulated arrays of 6 stem cells, to represent intestinal crypts, and 120 stem cells, to represent the corneal limbus (Fig. 6A,B). Total stem cell numbers remained unchanged during our simulations of stem cell replacement, so only one stem cell population could be lost. For arrays of 6 randomly distributed stem cells, mosaicism was lost within 200 stem cell generations in all simulations of 10% and 50% stem cell replacement (Fig. 6A). The median survival of mosaicism was respectively 38 and 7 stem cell generations, for 10% and 50% replacement. In contrast, for arrays of 120 randomly distributed stem cells, the median survival of mosaicism was >1000 stem cell generations for both 10% and 50% stem cell replacement (Fig. 6B). This figure is unlikely to be exceeded in many tissues during the lifespan of a laboratory mouse.

To further investigate how array size affected loss of mosaicism, we simulated 50% LESC replacement, in arrays with 30–360 LESCs, over 1000 LESC generations. This confirmed that mosaicism was more easily lost in smaller arrays (Fig. 6C–F). Furthermore, differences in frequency of loss of mosaicism among array sizes were greater and more significant than those between balanced and unbalanced arrays (Fig. S19) or between random and clumped arrays (Fig. S20). In most cases the median survival of mosaicism was >1000 LESC generations for arrays of 120 or 360 LESCs, which is the range that we predicted for the mouse limbus. However, for arrays with only 30 LESCs, the median survival of mosaicism was <200 LESC generations.

## 4 Discussion

### 4.1 Evidence that stochastic neutral drift could occur among mouse LESCs

Our main conclusion is that the computer model supports the hypothesis that stochastic LESC replacement by neighbouring LESC lineages, leading to neutral drift, without changing the number of active LESCs, could account for the age-related changes demonstrated for corneal epithelial stripe patterns in mosaic and chimaeric mice (Collinson et al., 2002; Mort et al., 2009).

**Fig. 7:**
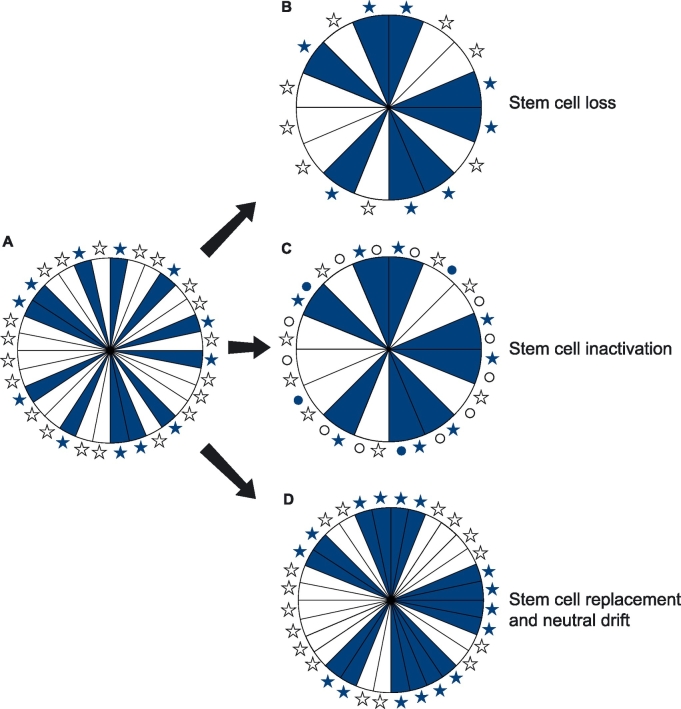
Decline in corneal epithelial stripe numbers with age may occur by loss, inactivation or replacement of limbal epithelial stem cells. Diagram showing mosaic corneal epithelia as disks with radial stripes, produced by active LESCs, represented by stars of the same colour, at the edge of the cornea. (A) Full complement of active LESCs, randomly distributed around the limbal circumference. (B-D) Alternative possibilities that could cause an age-related decline in corneal epithelial stripe numbers: (B) loss of some LESCs, (C) inactivation of some LESCs (shown as small circles instead of stars), (D) replacement of some LESCs leading to neutral drift of LESC populations. Modified after Mort et al., 2012.

Although simulated LESC replacement produced a similar decline in the corrected stripe number to the biological results, with a rapid early decline, which slowed later, this does not exclude other possibilities. For example, loss or irreversible inactivation of LESCs, without replacement, might be restricted to younger mice. A combination of LESC replacement plus additional LESC loss (or inactivation) without replacement could also occur. We, therefore, conclude that the biological observations could be accounted for by any combination of (1) progressive LESC loss without replacement, (2) progressive LESC inactivation without replacement and (3) stochastic LESC replacement leading to neutral drift, as shown in Fig. 7. No quantitative conclusions can be drawn about the likely biological frequency of any of these possible mechanisms from the computer simulations because there are too many unknown variables in the biological system. Although there is some evidence that human LESC numbers and/or activity may decline with age (Notara et al., 2013), at present we have no data that distinguish among these three possibilities. If some LESCs divide symmetrically to produce two TACs or two LESCs, this may often result in stochastic LESC replacement. However, symmetric division would be insufficient evidence that stem cell replacement occurs because symmetric division to two TACs could also occur if some LESCs were lost without replacement. Other evidence, such as lineage tracing, would be required to distinguish between these possibilities.

In mouse lineage-tracing experiments, some labelled clones, which extended across the radius of the corneal epithelium, were discontinuous and/or disconnected from the limbus (Dorà et al., 2015; Richardson et al., 2017). Dorà et al. (2015) suggested that this could be explained if LESCs cycled through periods of activity and quiescence or if some labelled LESCs were lost or replaced by unlabelled neighbouring LESCs, leading to stochastic neutral drift. In some cases, the peripheral end of a disconnected stripe was radially aligned with a small, labelled region in the limbus (Dorà et al., 2015). This was consistent with a previously active LESC becoming quiescent but remaining in the limbus, so it favoured the possibility that LESCs showed intermittent periods of activity and quiescence. However, not all disconnected stripes were aligned with labelling in the limbus and the frequency of this relationship was not quantified. Thus, it is possible that intermittent LESC quiescence, LESC loss (or irreversible inactivation) without replacement and LESC replacement all occur in the mouse corneal limbus.

Further lineage-tracing studies could be designed to investigate whether LESC loss or replacement predominates in the mouse corneal limbus. Unlike the chimaeras and X-inactivation mosaics, one lineage-tracing experiment with an inducible CAGG-CreER;R26-*LacZ* marker showed no clear trend for corneal stripe widths to increase with chase times between 6 and 20 weeks, after inducing labelling at 12 weeks (Dorà et al., 2015). However, another lineage-tracing study, using multi-coloured K14-CreER^T2^;R26-confetti mice, showed convincingly that, following labelling at 6 weeks, stripes coarsened as the mice aged from 16 weeks to 60 weeks (Richardson et al., 2017). This approach could be extended to help distinguish between LESC replacement and loss if stripe widths were compared after initiating labelling at different ages and chasing for various times. If stripe widths depended on age, regardless of when labelling was initiated, this would argue that LESC loss (or irreversible inactivation) was the main cause of the coarsening of the stripe pattern. If, however, stripe widths depended on chase times, regardless of age at labelling, this would argue that LESC replacement, leading to neutral drift, was the major mechanism. To our knowledge, no lineage-tracing experiment has yet provided this type of information for the corneal epithelium, even though stripe patterns were induced at different ages in one study (Dorà et al., 2015). Long-term, real-time imaging with the multi-coloured R26-confetti lineage marker (Di Girolamo et al., 2015; Richardson et al., 2017) might be particularly useful for this type of investigation.

### 4.2 Limitations of the computer model

Our model assumed that LESCs are arranged in a 1-dimensional ring and each LESC could only be replaced by either of its two adjacent LESC neighbours. However, the mouse limbus is a narrow 2-dimensional annulus, so many LESCs could have more than two adjacent LESC neighbours. This might increase the frequency of LESC replacement. LESCs might also be clumped together into larger coherent clones, so the corrected stripe number might decline more slowly, as in the simulated 1-dimensional clumped arrays. However, such quantitative effects would only affect the scale of the simulations. They would not undermine the main conclusion that stochastic LESC replacement could explain the observed age-related decline in corrected stripe numbers in the corneas of mosaic and chimaeric mice.

Although there is no evidence for an uneven distribution of stripes around the corneal circumference in mosaic mice (Mort et al., 2009), two studies suggest that mouse LESCs may be distributed unevenly around the limbus (Pajoohesh-Ganji et al., 2006; Zhao et al., 2009). Our simulation did not allow for this possibility but, again, it would not undermine the main conclusion.

### 4.3 Relevance of computer simulations to other tissues

Although the computer model was designed specifically for the corneal epithelium, our results may shed some light on why age had no significant effect on mosaic patterns in the adrenal cortex (Chang et al., 2011) yet it had a more extreme effect on intestinal crypt mosaicism, culminating in loss of mosaicism (Schmidt et al., 1988).

The adult adrenal cortex is maintained by stem cells near the periphery (reviewed by Lerario et al., 2017) and histological sections of adrenal cortices from chimaeric or mosaic mice display a pattern of radial stripes (Iannaccone and Weinberg, 1987; Morley et al., 1996), which is similar to the radial stripes in the corneal epithelium. In contrast to the corneal epithelium, however, the corrected stripe number in the adrenal cortex of mosaic transgenic mice showed no significant age-related decline between 8 and 52 weeks (Chang et al., 2011). It is possible that stem cell loss, inactivation or replacement does not occur or is much slower in the adrenal cortex. For example, stem cells probably divide slowly in the adrenal cortex and other tissues with slow turnover rates under normal physiological conditions (Rando, 2006). Also, adrenocortical stem cells are distributed within a quasi 2-dimensional outer ‘shell’ of the cortex, so they may be arranged in larger coherent clones than in the quasi 1-dimensional limbal ring. Our simulation results imply that larger coherent clonal groups would probably both delay and slow the reduction in corrected stripe numbers. Thus, even if stem cell loss and/or replacement occurred at a comparable frequency among adrenocortical stem cells and LESCs, the effect on the stripe pattern in the adrenal cortex might be significantly less.

Our simulations imply that, if intestinal crypts each have only 5–7 active stem cells (Kozar et al., 2013), loss of mosaicism would take far fewer stem cell generations than in the corneal epithelium. This, together with the short intestinal crypt stem cell cycle time (Barker et al., 2008), helps explain how loss of mosaicism within crypts can occur by two weeks after birth.

## 5 Conclusions

The computer simulation study showed that the decline in coherent clones of LESCs observed in ageing mouse eyes could be explained by LESC replacement leading to stochastic neutral drift without reducing absolute LESC numbers. This shows that stochastic neutral clonal drift of LESCs could be a feature of normal homeostasis of the mouse corneal epithelium. We do not exclude the possibility of progressive LESC loss or inactivation. All three mechanisms may be acting and their relative contribution to corneal epithelial maintenance in the adult mouse deserves further investigation.

## Appendix A Supplementary data

Supplementary material Image 1
