# Supplementary material for: Computer simulation of neutral drift among limbal epithelial stem cells of mosaic mice
Source: Stem Cell Res. 2018 Jul;30:1–11. doi: 10.1016/j.scr.2018.05.003 (PMC6049397; doi:10.1016/j.scr.2018.05.003)
Supplement: Supplementary file 1 — Supplementary material [file mmc1.pdf]

Computer simulation of neutral drift among limbal epithelial stem cells of mosaic mice

John D. West, Richard L. Mort, Robert E. Hill, Steven D. Morley and J. Martin Collinson

SUPPLEMENTARY FIGURES & TABLES

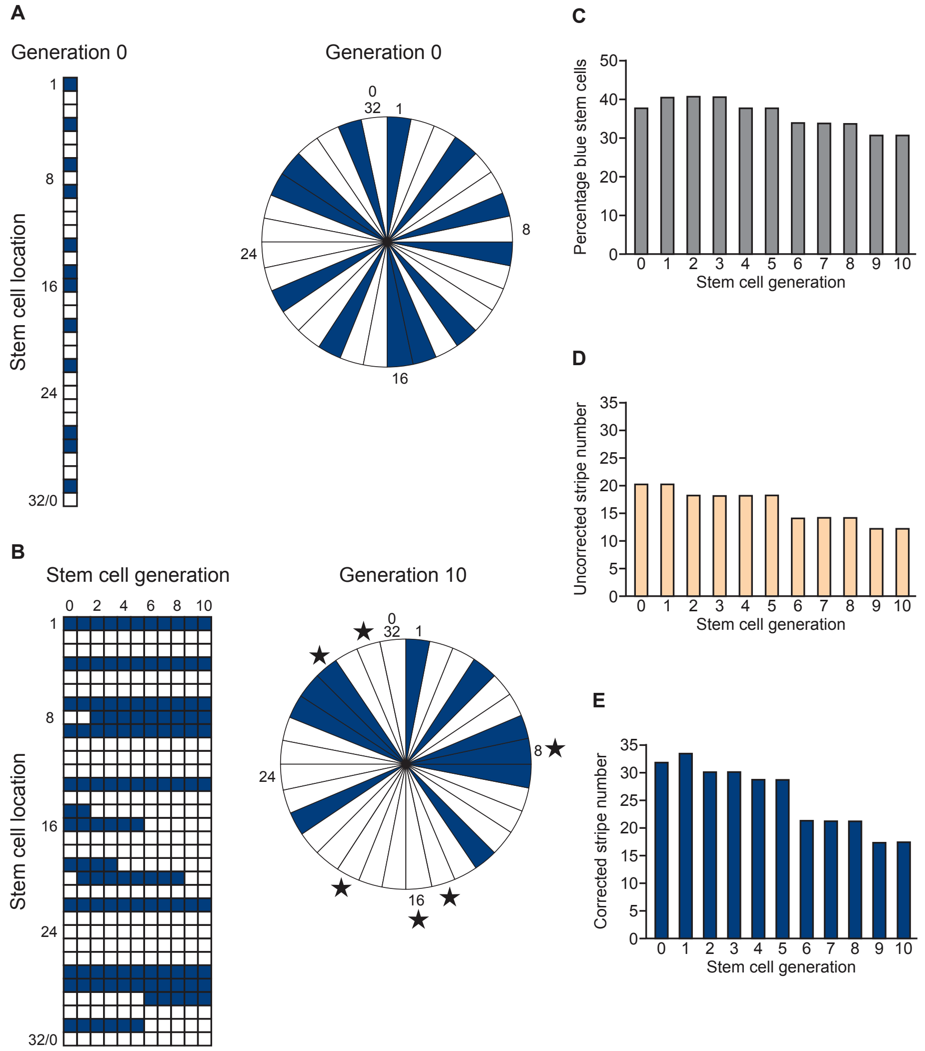

Figure S1. Interpretation of computer simulation of stochastic limbal epithelial stem cell replacement in random distributions of blue and white LESCs in a mosaic corneal epithelium **(A)** The column of blue (positively labelled) and white (unlabelled) squares represents a simulation of 12 blue and 20 white LESCs (37.5% blue) arranged randomly in a vertical array that forms a simulated circle, so the LESC at position 32 is between positions 31 and 1 (so is labelled 32/0). This column represents LESCs arranged in a mosaic, circular limbus at generation 0 (G0). The circular diagram is an interpretation of the vertical array of LESCs, representing a mosaic corneal epithelium, where the LESCs reside at the periphery and produce clones of transient amplifying cells (TACs), which migrate centripetally towards the centre so forming radial stripes of blue or white cells according to the LESC population. The circular tissue is divided into 32 sectors each of which represents a region maintained by a radial clone of cells produced by the LESC at the periphery. The distribution of blue and white LESCs at the periphery matches that shown in the vertical array on the left. The striped pattern at the beginning of the simulation (G0) has 12 blue LESCs, forming 10 blue stripes, and 20 white LESCs, forming 10 white stripes. **(B)** The diagram on the left shows 11 columns of blue and white LESCs. Each column represents a new LESC generation from G0 on the left to G10 on the right. At each generation an LESC may survive or be replaced by an LESC of the same colour as one of its adjacent neighbours by a stochastic event but only some replacements change the colour of the LESCs. Results are shown for a simulation where each LESC had a 10% chance of being replaced at each LESC division and the display extends to the right at each successive generation. The radial striped patterns on the right show the pattern produced by the arrangement of LESCs at G10. The black stars indicate where the LESCs at G0 have been replaced by LESCs of the other colour at G10. **(C)** The percentage of blue LESCs at each LESC generation. **(D)** The uncorrected stripe number (blue plus white stripes) at each LESC generation. **(E)** The corrected stripe number at each LESC generation.

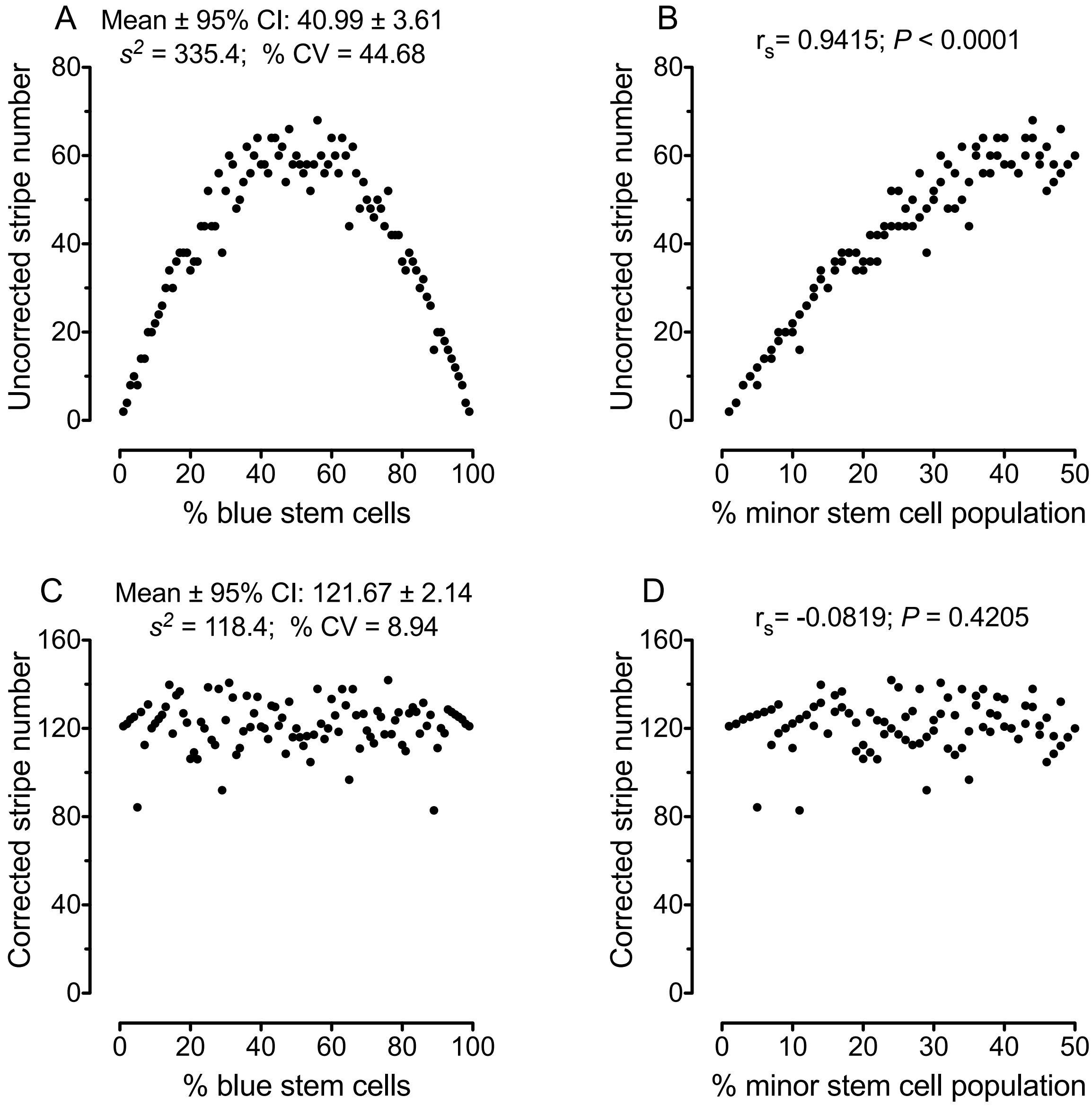

Figure S2. Distributions of uncorrected and corrected stripe numbers in simulations with different proportions of blue and white limbal epithelial LESCs (LESCs)
Random distributions of 120 LESCs were simulated comprising 1 to 99% blue LESCs. These represent LESCs that would produce radial stripes in the corneal epithelium. **(A, B)** The uncorrected stripe number varies with the percentage of blue LESCs in the array (A) and is highly correlated with the percentage of the minor population of LESCs (blue or white) in the array (B). **(C, D)** The corrected stripe number corrects for the expected number of adjacent LESCs of the same population and provides an estimate of the number of coherent clones of LESCs in the array. In this case the LESCs were arranged randomly so the expected number of coherent LESC clones is the same as the number of LESCs (120). The observed mean corrected stripe number from the simulations of 121.7 is close to the expected value of 120. The corrected stripe number in (C) shows less variation (smaller variance and percentage coefficient of variation) than the uncorrected stripe number in (A) and unlike the uncorrected stripe number in (B) it is not significantly correlated with the percentage of the minor population of LESCs in the array (D). Abbreviations: % CV, percentage coefficient of variation; r_s, Spearman's correlation coefficient; s^2, variance. *P*-values for Spearman's correlation are shown in B and D.

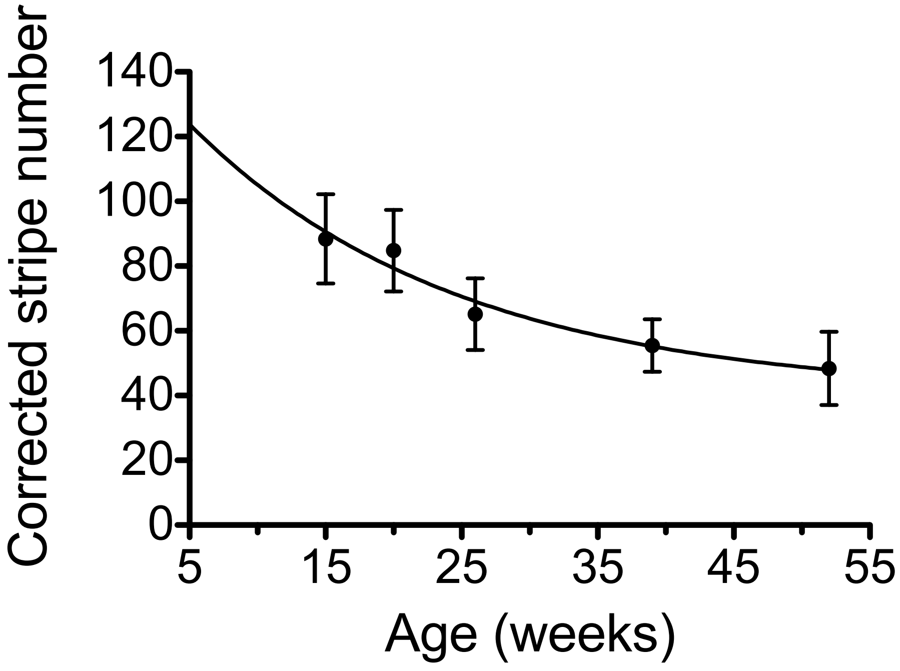

Figure S3. Age-related decline in corrected corneal epithelial stripe number
Corneal epithelial corrected stripe numbers (± 95% CI), shown in Fig. 1B, re-plotted starting at 5 weeks which is the age when the stripes appear to first emerge (Collinson *et al*., 2002). The data approximate a one-phase exponential decay and predicts a corrected stripe number of 123.8 at five weeks, which declines to approximately half this number after 26 weeks (at 31 weeks of age).

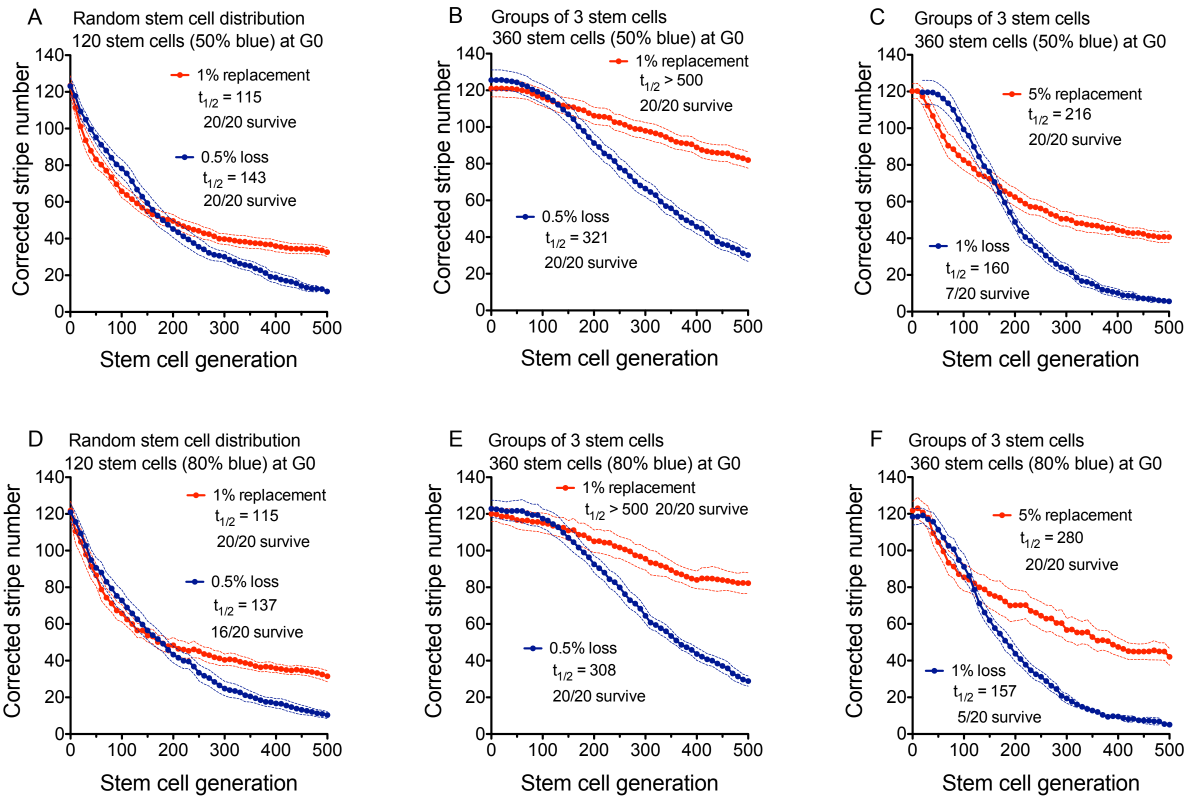

Figure S4. Decline in corrected stripe number in simulations with low probabilities of limbal epithelial stem cell loss or replacement

The effects of LESC loss or replacement on the corrected stripe number (mean ±95% CI) are shown over 500 LESC generations for 12 sets of 20 simulations for arrays initiated with 50% blue LESCs (A-C) or 80% blue LESCs (D-F). Data were plotted for every tenth generation. **(A, D)** 0.5% LESC loss or 1% LESC replacement in simulations of 120 LESCs that were randomly distributed at generation 0 (G0). **(B, E)** 0.5% LESC loss or 1% LESC replacement in simulations of 360 LESCs in clumped arrays of groups of three (random distributions of groups of three same-coloured LESCs, rather than random distributions of single LESCs) at G0. **(C, F)** 1% LESC loss or 5% LESC replacement in simulations of 360 LESCs in clumped arrays of groups of three LESCs at G0. Corrected stripe number half-lives are shown as $t_{1/2}$ LESC generations and were determined for the mean corrected stripe number of the set of 20 simulations as described in the Materials and Methods. The frequency of simulations where both cell populations survived for 500 LESC generations is shown after the half-life. As the corrected stripe number is not calculated if either LESC population is lost (see Materials and Methods), the mean corrected stripe number is based on less than 20 simulations in later cell generations of the cell loss simulations shown in (C), (D) and (F). The comparisons shown here are similar to those shown in Fig. 4 except that, here, the probabilities of LESC loss or replacement are lower so the corrected stripe numbers decline more slowly (with longer corrected stripe number half-lives).

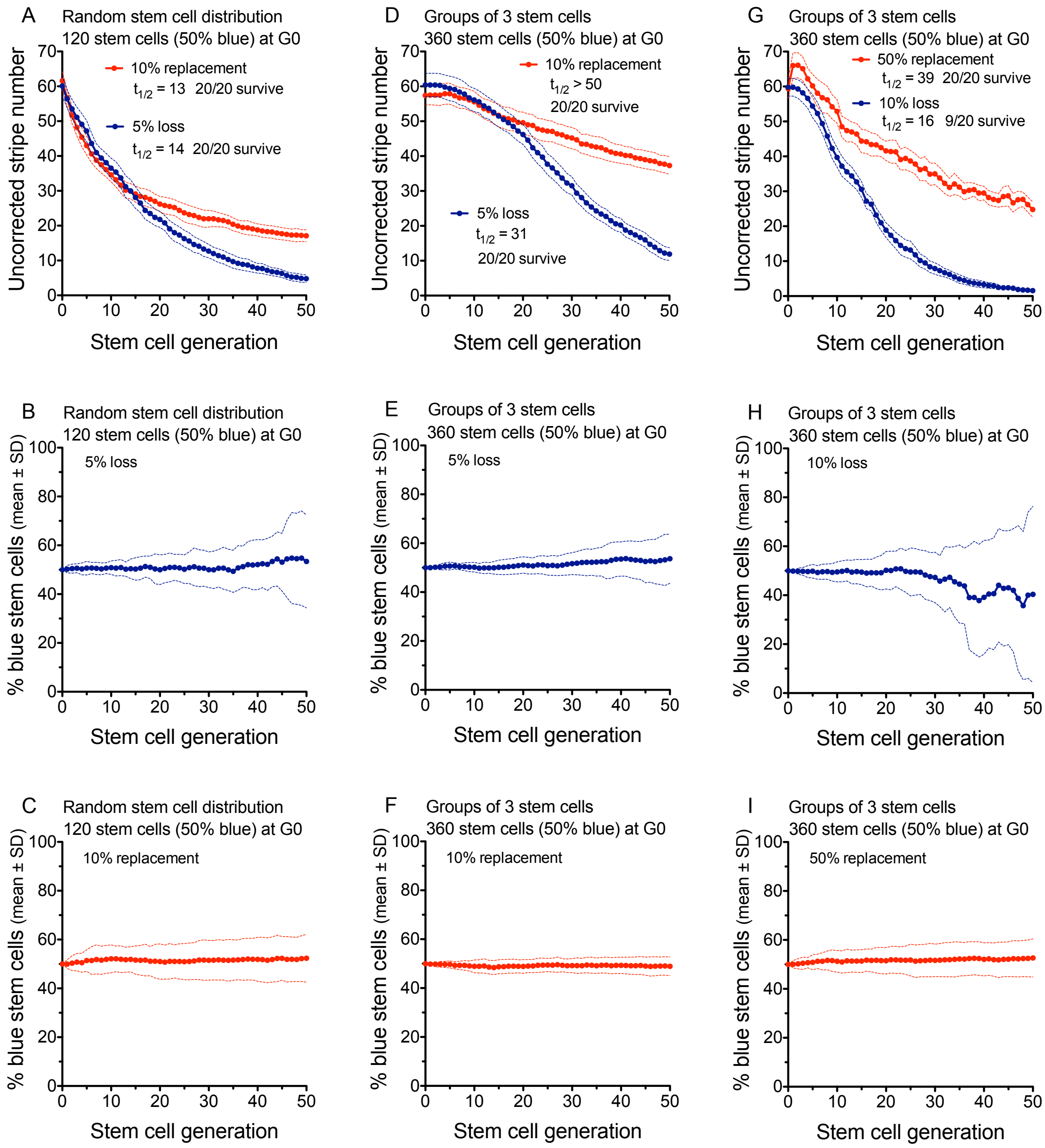

Figure S5. Changes in uncorrected stripe number and percentage blue LESCs in simulations of LESC loss or LESC replacement for arrays initiated with 50% blue LESCs

Changes in the mean (± 95% CI) uncorrected stripe number and the mean (± SD) percentage of blue LESCs are shown for sets of 20 simulations over 50 LESC generations. **(A-C)** Effects of 5% LESC loss and 10% LESC replacement for simulations of 120 LESCs that were randomly distributed at G0. **(D-F)** Effects of 5% LESC loss or 10% LESC replacement for simulations of clumped arrays with 360 LESCs that were arranged in groups of three at G0. **(G-I)** Effects of 10% LESC loss or 50% LESC replacement for simulations of clumped arrays with 360 LESCs that were arranged in groups of three at G0. The corrected stripe number half-life is shown as $t_{1/2}$ LESC generations (in A, D and G) and was determined for the mean corrected stripe number for the set of 20 simulations as described in the Materials and Methods. The frequency of simulations where both LESC populations survived for 50 LESC generations is shown after the half-life. Abbreviation: SD, standard deviation.

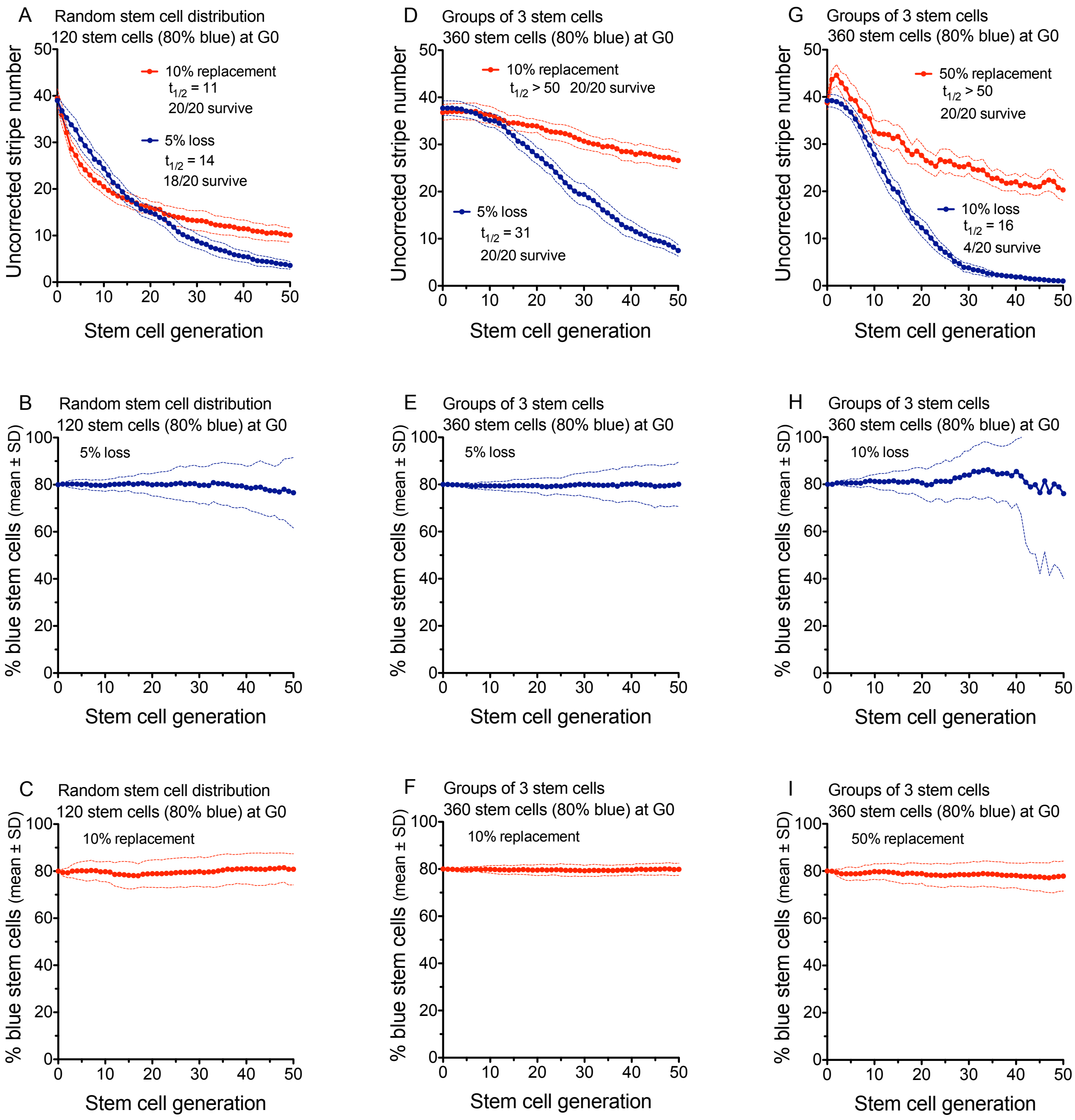

Figure S6. Changes in uncorrected stripe number and percentage blue LESCs in simulations of LESC loss or LESC replacement for arrays initiated with 80% blue LESCs
Changes in the mean (± 95% CI) uncorrected stripe number and the mean (± SD) percentage of blue LESCs are shown for sets of 20 simulations over 50 LESC generations. **(A-C)** Effects of 5% LESC loss and 10% LESC replacement for simulations of 120 LESCs that were randomly distributed at G0. **(D-F)** Effects of 5% LESC loss or 10% LESC replacement for simulations of clumped arrays with 360 LESCs that were arranged in groups of three at G0. **(G-I)** Effects of 10% LESC loss or 50% LESC replacement for simulations of clumped arrays with 360 LESCs that were arranged in groups of three at G0. The corrected stripe number half-life is shown as $t_{1/2}$ LESC generations (in A, D and G) and was determined for the mean corrected stripe number for the set of 20 simulations as described in the Materials and Methods. The frequency of simulations where both LESC populations survived for 50 LESC generations is shown after the half-life. Abbreviation: SD, standard deviation.

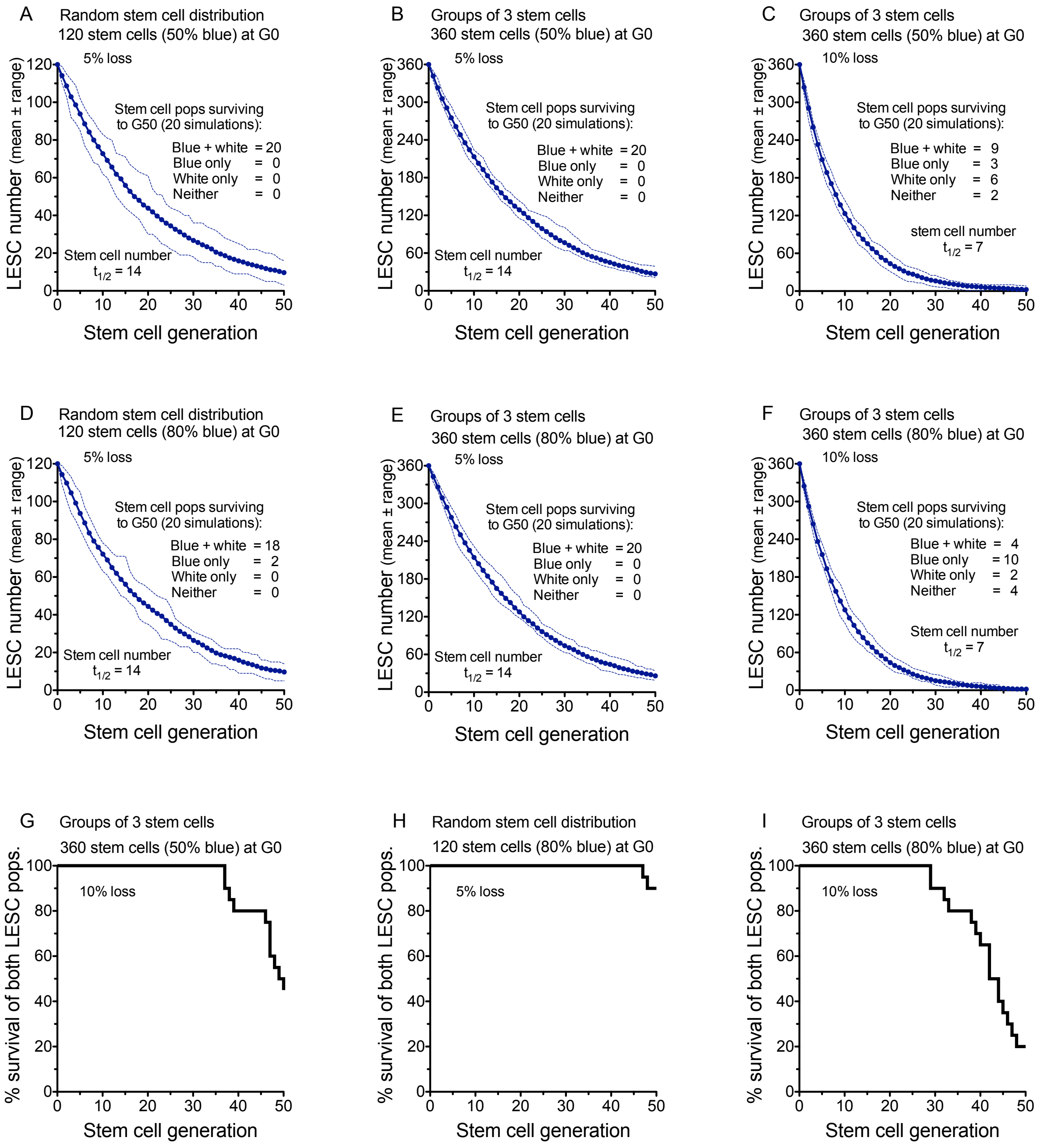

Figure S7. Changes in cell numbers for limbal epithelial stem cell loss simulations for arrays initiated with 50% or 80% blue LESCs

(A-F) Decline in LESC numbers (mean ± range) in simulations with a probability of LESC loss per LESC per generation of either 5% (A, B, D, E) or 10% (C, F). The half-life is shown as $t_{1/2}$ LESC generations and was defined as explained in the Materials and Methods. **(G-I)** Survival of both LESC populations in the three array sets shown in (C), (D) and (F), where one or both LESC populations were lost before LESC generation 50. Abbreviation: G50, stem cell generation 50; LESC, limbal epithelial stem cell; pop, population.

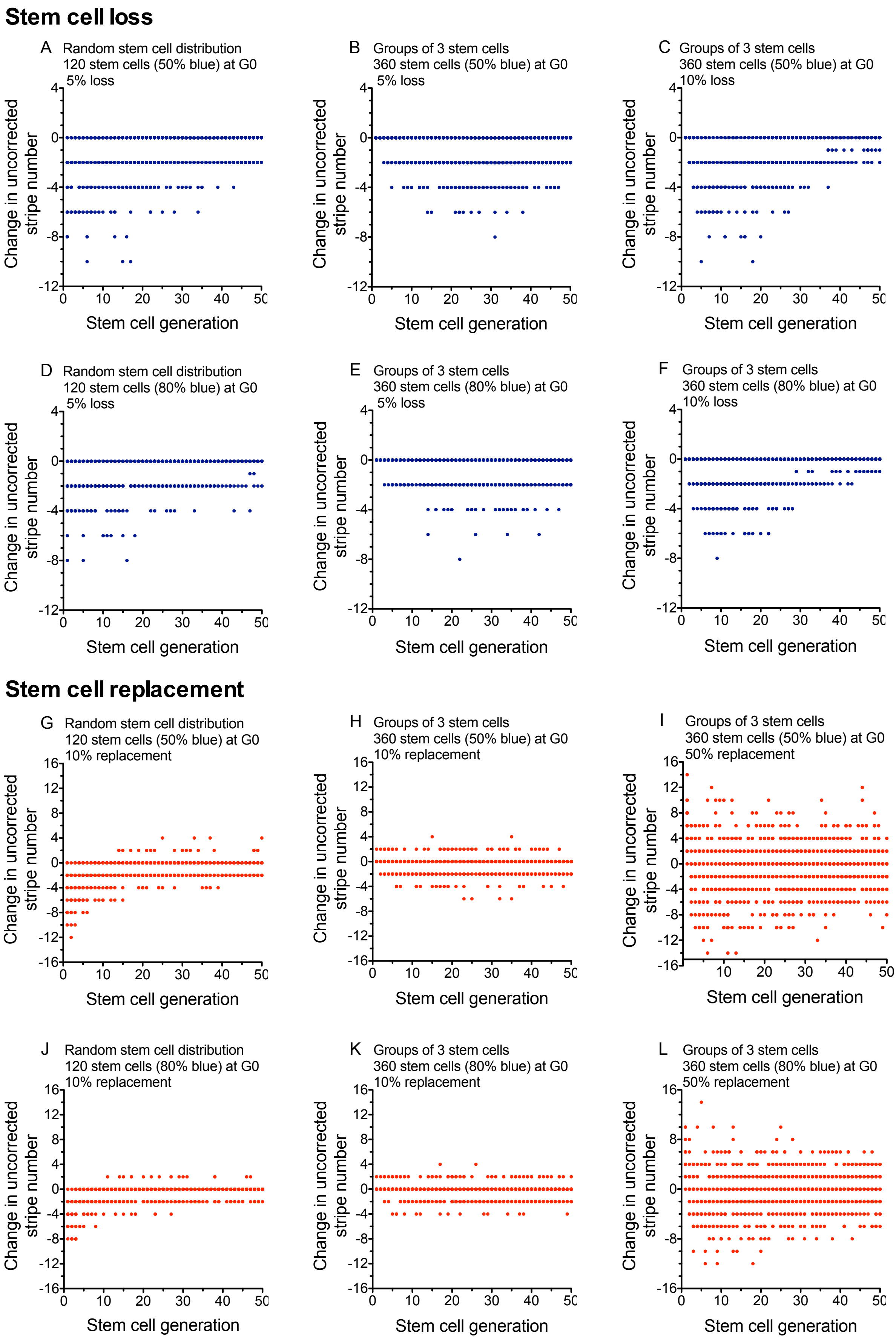

Figure S8. (See legend on the next page.)

(See Figure S8 on the previous page.)

Figure S8. Changes in uncorrected stripe numbers after each limbal epithelial stem cell division for arrays initiated with 50% or 80% blue LESCs
(A-F) Changes in uncorrected stripe numbers after each division in arrays that initially had 50% (A-C) or 80% (D-F) blue LESCs and then underwent LESC loss. (A, D) Random distributions of 120 LESCs with 5% LESC loss per generation. (B, E) Clumped distributions of 360 LESCs, arranged in groups of three, with 5% LESC loss per generation. (C, F) Clumped distributions of 360 LESCs, arranged in groups of three, with 10% LESC loss per generation. In most cases, LESC loss caused the uncorrected stripe number to remain unchanged or decline by a multiple of two because deletion of a stripe also caused the two flanking stripes to merge. However, in (C), (D) and (F) one LESC population was lost and the uncorrected stripe number declined by only one, when the uncorrected stripe number declined from two stripes to one. **(G-L)** Changes in uncorrected stripe numbers after each division in arrays that initially had 50% (G-I) or 80% (J-L) blue LESCs and then underwent LESC replacement. (G, J) Random distributions of 120 LESCs with 10% LESC replacement per generation. (H, K) Clumped distributions of 360 LESCs, arranged in groups of three, with 10% LESC replacement per generation. (I, J) Clumped distributions of 360 LESCs, arranged in groups of three, with 50% LESC replacement per generation. Twenty simulations were run for each array set so there were 20 arrays per LESC division but some values are superimposed in the graphs.

Stem cell loss

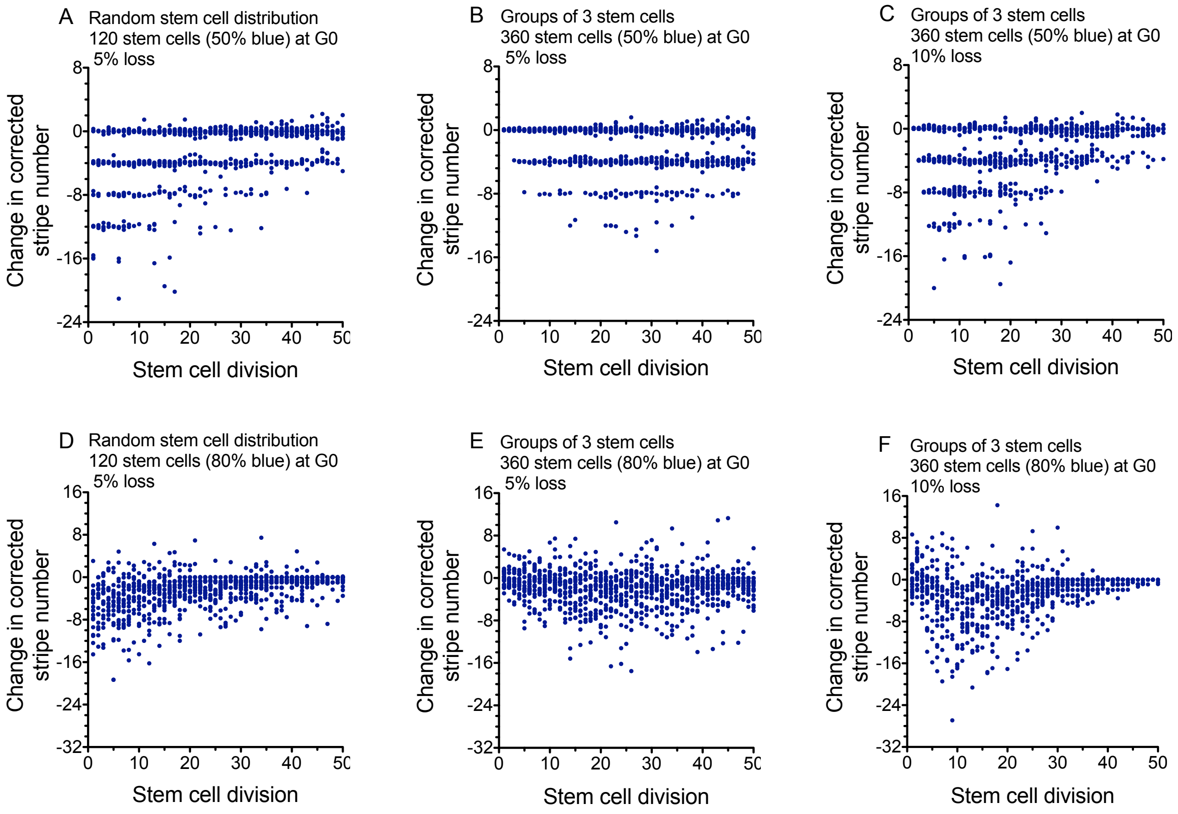

Stem cell replacement

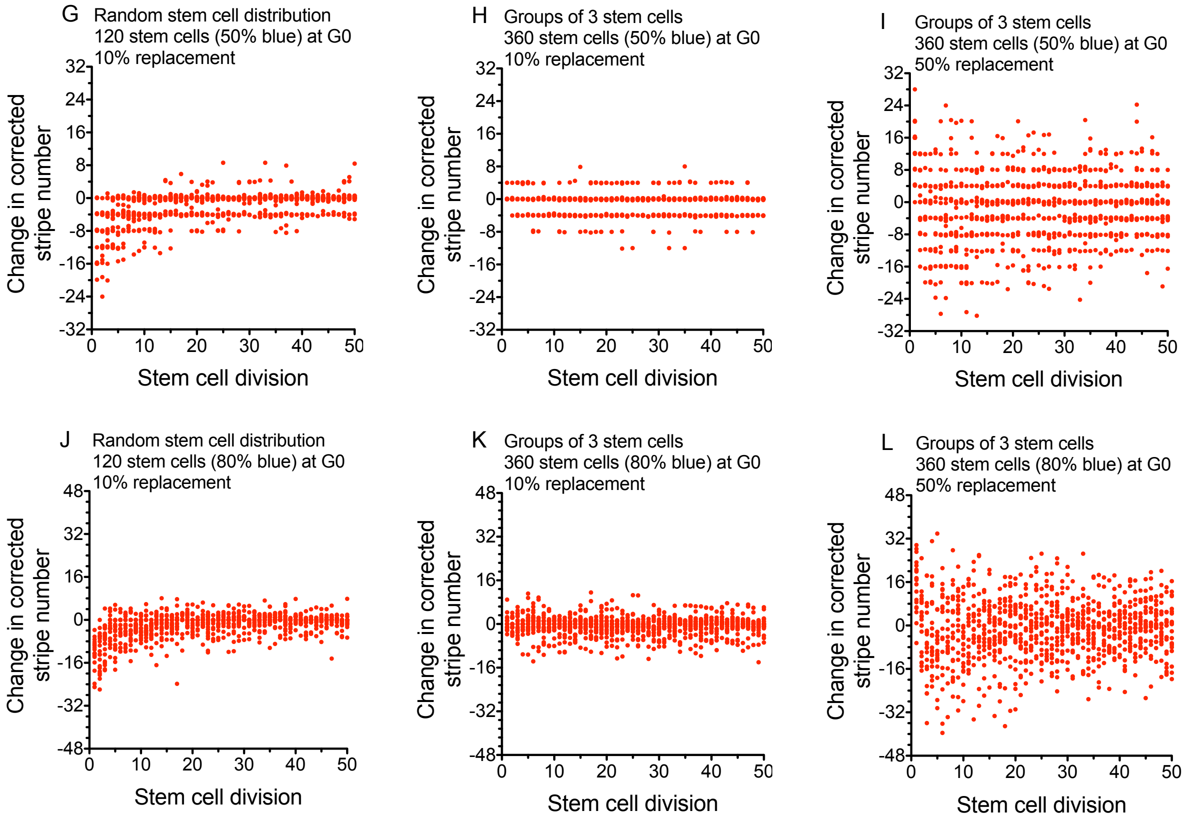

Figure S9. (See legend on the next page.)

(See Figure S9 on the previous page.)

Figure S9. Changes in corrected stripe numbers after each limbal epithelial stem cell division for arrays initiated with 50% or 80% blue LESCs

(A-F) Changes in corrected stripe numbers after each division in arrays that initially had 50% (A-C) or 80% (D-F) blue LESCs and then underwent LESC loss. (A, D) Random distributions of 120 LESCs with 5% LESC loss per generation. (B, E) Clumped distributions of 360 LESCs, arranged in groups of three, with 5% LESC loss per generation. (C, F) Clumped distributions of 360 LESCs, arranged as randomly distributed groups of three LESCs, with 10% LESC loss per generation. **(G-L)** Changes in corrected stripe numbers after each division in arrays that initially had 50% (G-I) or 80% (J-L) blue LESCs and then underwent LESC replacement. (G, J) Random distributions of 120 LESCs with 10% LESC replacement per generation. (H, K) Clumped distributions of 360 LESCs, arranged in groups of three, with 10% LESC replacement per generation. (I, J) Clumped distributions of 360 LESCs, arranged in groups of three, with 50% LESC replacement per generation. Twenty simulations were run for each array set so there were 20 arrays per LESC division but some values are superimposed in the graphs.

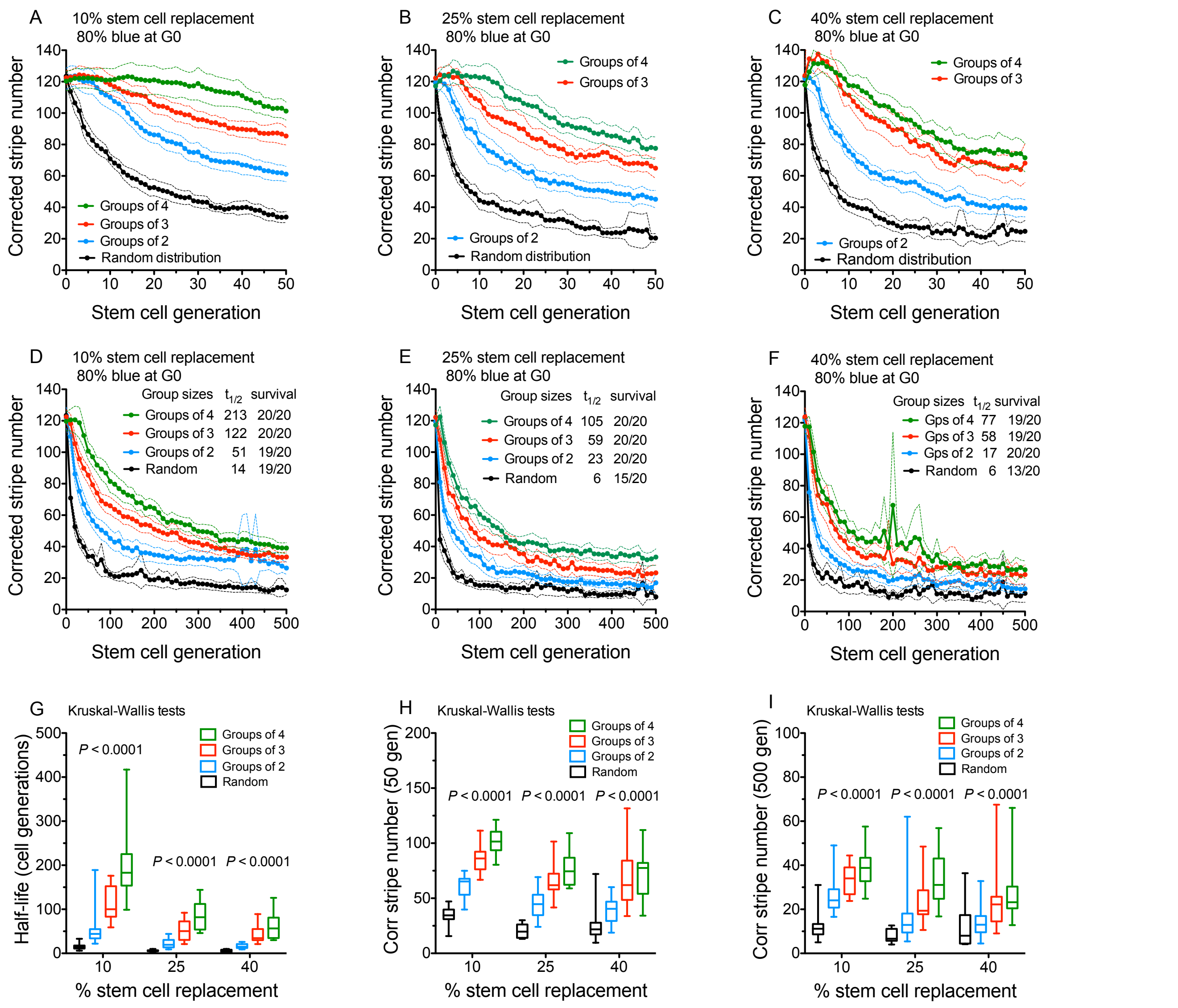

Figure S10. Effect of initial limbal epithelial stem cell distribution on the decline in corrected stripe number in simulations of LESC replacement in arrays with 80% blue LESCs at G0
(A-F) Comparison of decline in corrected stripe number (mean ± 95% CI; 20 simulations per set) over 50 (A-C) and 500 (D-F) LESC generations for four different types of array distributions with 80% blue LESCs at G0. Random arrays had 120 LESCs and clumped arrays with groups of 2, 3 or 4 LESCs had 240, 360 and 480 LESCs respectively. These array types were compared for simulations of 10% (A, D), 25% (B, E) and 40% (C, F) LESC replacement. In (D-F), data were plotted for every tenth generation and "survival" indicates the frequency of simulations where both LESC populations survived for 500 LESC generations. Loss of the minor cell population was often preceded by survival, for a few cell generations, of a single stripe that was only one cell thick. This produced an abnormally high corrected stripe number so the set of simulations had a large 95% confidence interval. **(G-I)** The corrected stripe number half-life (G), corrected stripe number at 50 LESC generations (H) and corrected stripe number at 500 LESC generations (I) were compared among the four different array distributions. The Kruskal-Wallis test was used because some results were not normally distributed (*P*-values are shown). Half-lives shown as $t_{1/2}$ LESC generations in (D-F) were defined as explained in the Materials and Methods and determined for the mean of the set of 20 simulations but half-lives in (G) were determined separately for each simulation. Box and whisker plots in (G-I) show the median (horizontal line within the box), upper and lower quartiles (top and bottom of boxes) and the minimum and maximum of all the data (ends of whiskers).

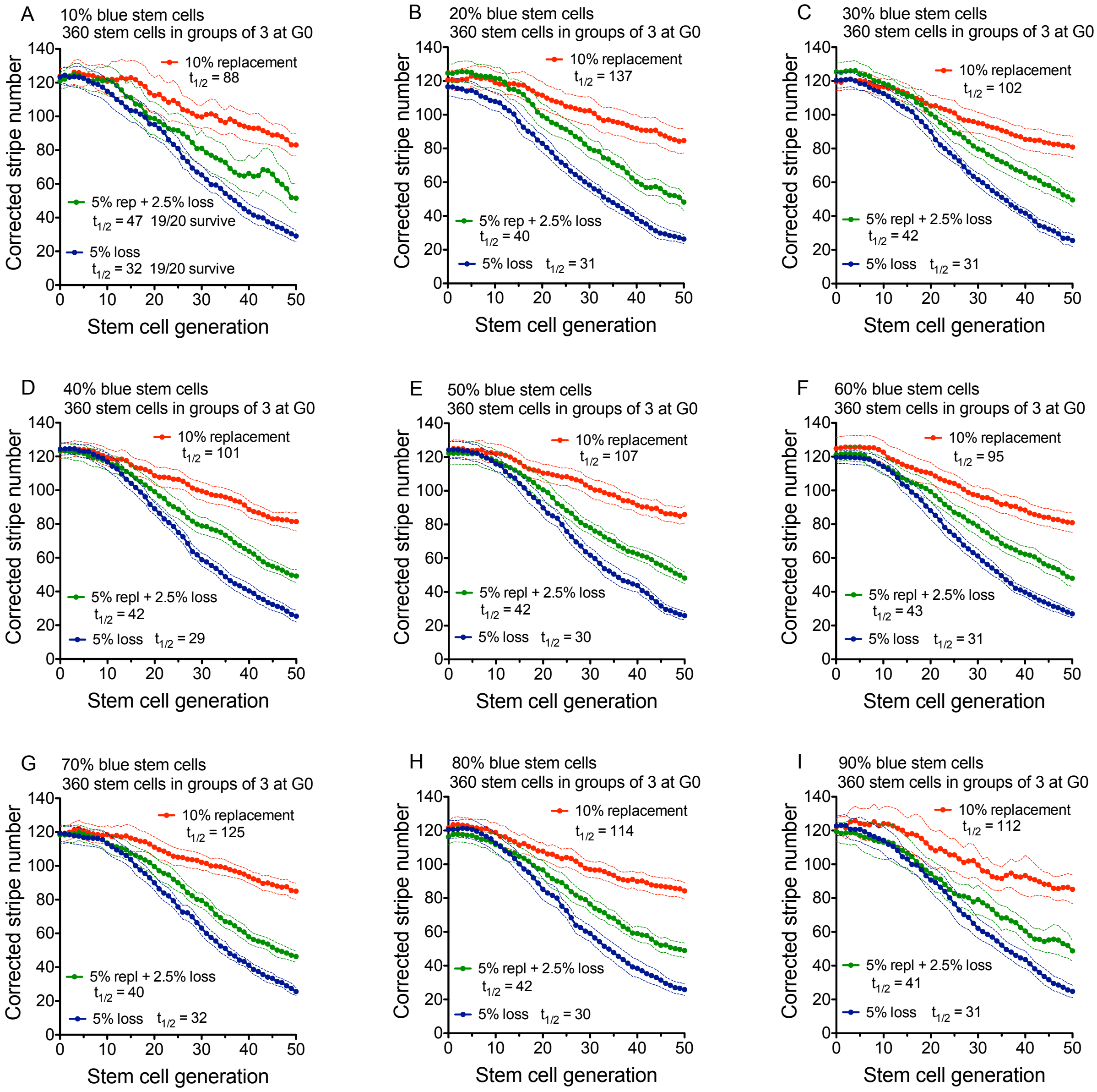

Figure S11. Effects of the initial percentage of blue LESCs and different combinations of LESC replacement and loss on the decline in corrected stripe numbers in simulations of clumped arrays

Decline in corrected stripe numbers (mean ± 95% CI; 20 simulations per set) over 50 LESC generations for clumped arrays with 360 LESCs, arranged in groups of three at G0. The effects of 10% LESC replacement, 5% LESC loss and a combination of 5% LESC replacement and 2.5% LESC loss were compared for simulations that had 10% (A), 20% (B), 30% (C), 40% (D), 50% (E), 60% (F), 70% (G), 80% (H) and 90% (I) blue LESCs at G0. Simulations were run for 500 LESC generations and the half-life ($t_{1/2}$) was defined as explained in the Materials and Methods. Both blue and white LESC populations survived for 50 LESC generations in each set of 20 simulations except for two of the sets of simulations with 10% blue LESCs at G0, shown in (A). For both the set of simulations with 5% LESC loss and the set with 5% LESC replacement plus 2.5% LESC loss, shown in (A), both LESC populations survived in 19/20 simulations.

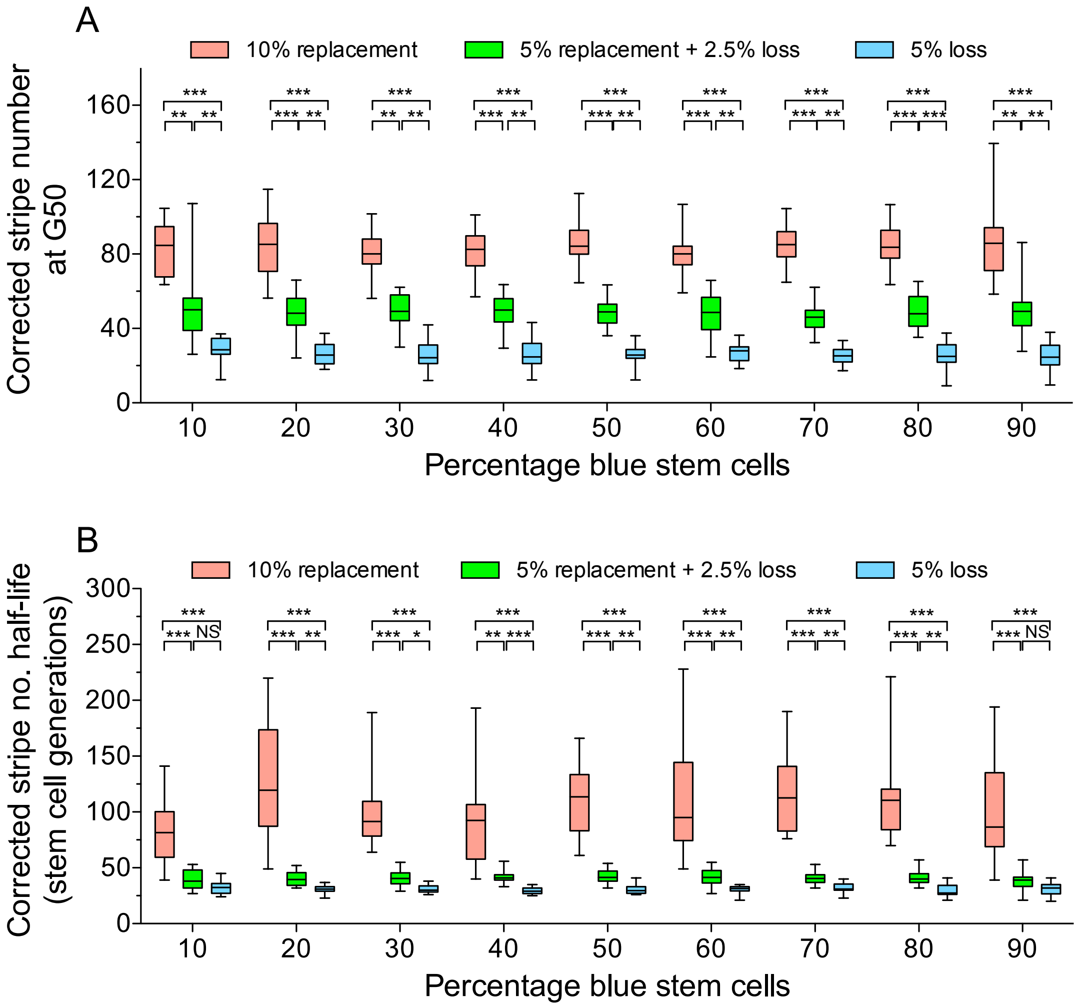

Figure S12. Evaluation of the effects of the initial percentage of blue LESCs and different combinations of LESC replacement and loss on the decline in corrected stripe numbers in simulations, shown in Figure S11.

(A) After 50 LESC generations, the corrected stripe number did not vary significantly among the nine initial percentages of blue LESCs for simulations of 10% LESC replacement (Kruskal-Wallis test; $P = 0.8643$), 5% LESC loss ($P = 0.4984$) or the combination of 5% LESC replacement and 2.5% LESC loss ($P = 0.9615$). However, it did vary among the three combinations of LESC replacement and loss ($P < 0.0001$ for each of the 9 percentages of blue LESCs) and results for pairwise comparisons by Dunn's multiple comparison test are shown by asterisks in the figure.

(B) The corrected stripe number half-life was determined separately for each simulation and did not vary significantly among the nine initial percentages of blue LESCs for simulations of 5% LESC loss ($P = 0.3388$) or the combination of 5% LESC replacement and 2.5% LESC loss ($P = 0.7016$). Although it did vary significantly among the nine percentages of blue LESCs for 10% LESC replacement ($P = 0.0111$), only simulations with 10% and 20% blue differed significantly by Dunn's multiple comparison test ($P < 0.05$). The corrected stripe number half-life varied among the three combinations of LESC replacement and loss ($P < 0.0001$ for each of the 9 percentages of blue LESCs) and results for pairwise comparisons by Dunn's multiple comparison test are shown by asterisks in the figure. * $P < 0.05$, ** $P < 0.01$, *** $P < 0.001$, NS = not significant. Box and whisker plots show the median (horizontal line within the box), upper and lower quartiles (top and bottom of boxes) and the minimum and maximum of all the data (ends of whiskers).

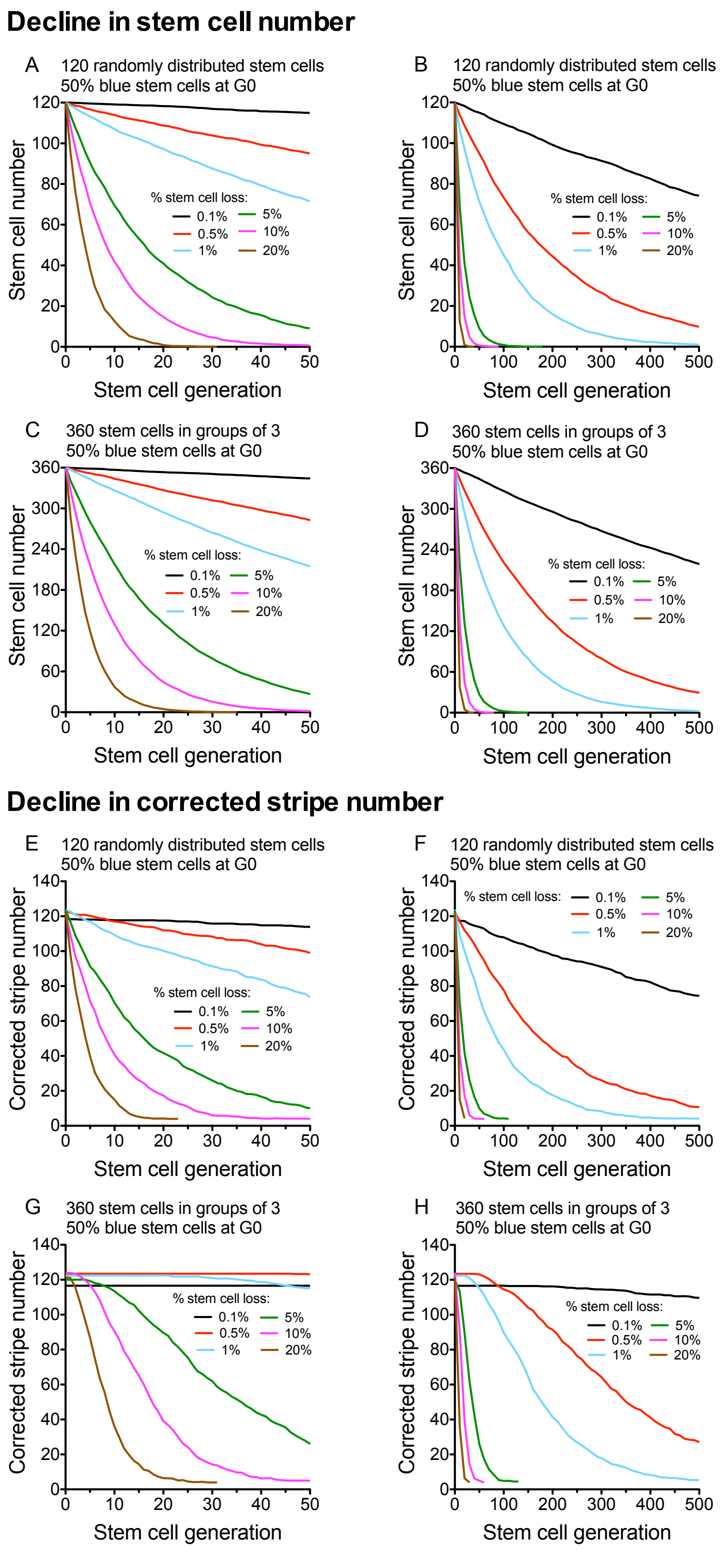

Figure S13. Effect of different probabilities of limbal epithelial stem cell loss on LESC number and corrected stripe number in arrays with 50% blue LESCs at G0
(A-D) Decline in mean LESC number, for simulations of arrays with 120 randomly distributed LESCs (A, B) and clumped arrays with 360 LESCs arranged in groups of three at G0 (C, D), shown for 50 (A, C) or 500 (B, D) LESC generations. **(E-H)** Decline in mean corrected stripe number for simulations of random arrays (E, F) and clumped arrays with groups of three LESCs (G, H) shown for 50 (E, G) or 500 (F, H) LESC generations. For clarity, error bars are not shown; 20 simulations per set.

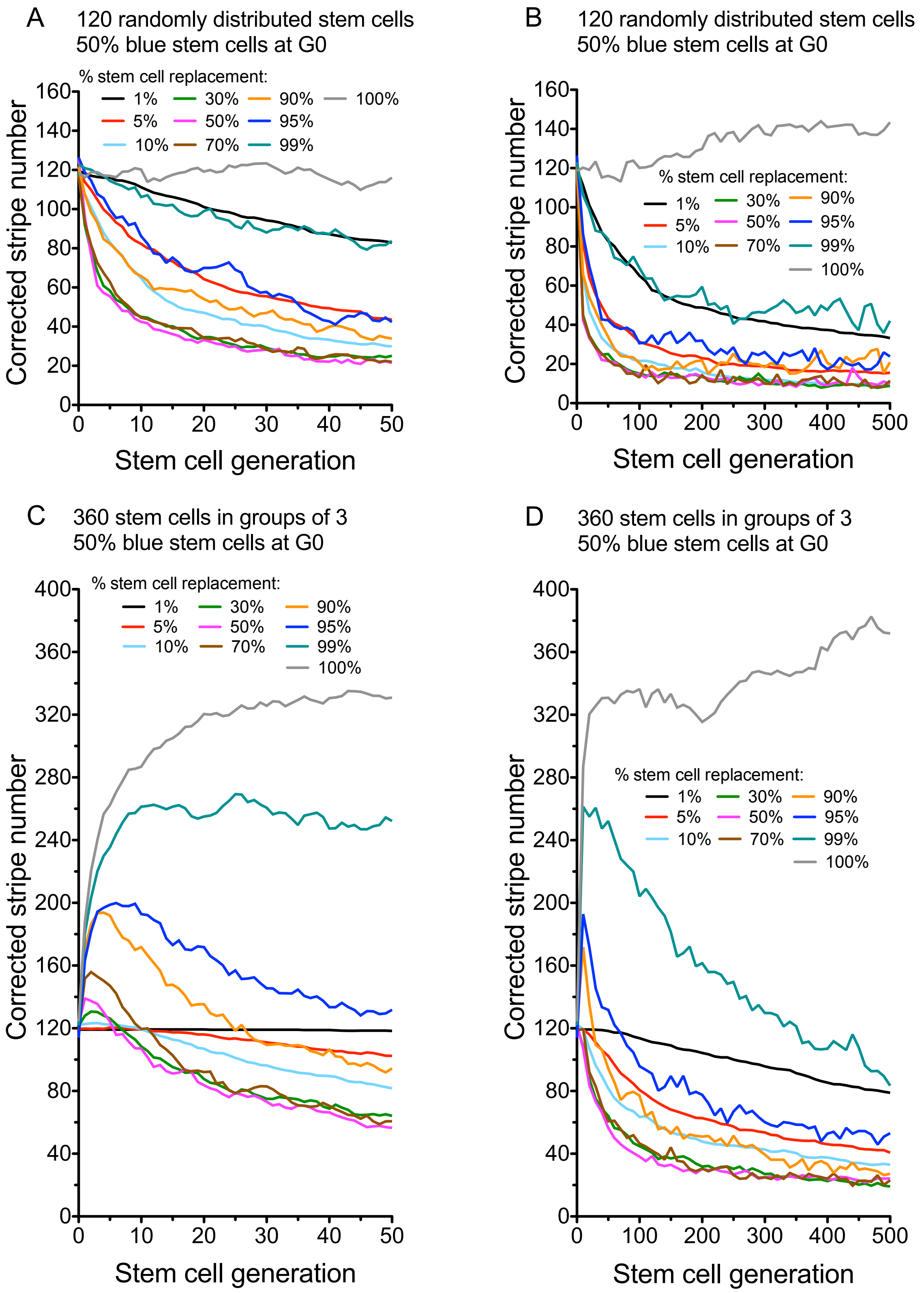

Figure S14. Effect of different probabilities of limbal epithelial stem cell replacement on corrected stripe number in arrays with 50% blue LESCs at G0

Decline in mean corrected stripe number, for simulations of arrays with 120 randomly distributed LESCs (A, B) and clumped arrays with 360 LESCs arranged in groups of three at G0 (C, D), shown for 50 (A, C) or 500 (B, D) LESC generations. For clarity, error bars are not shown; 20 simulations per set.

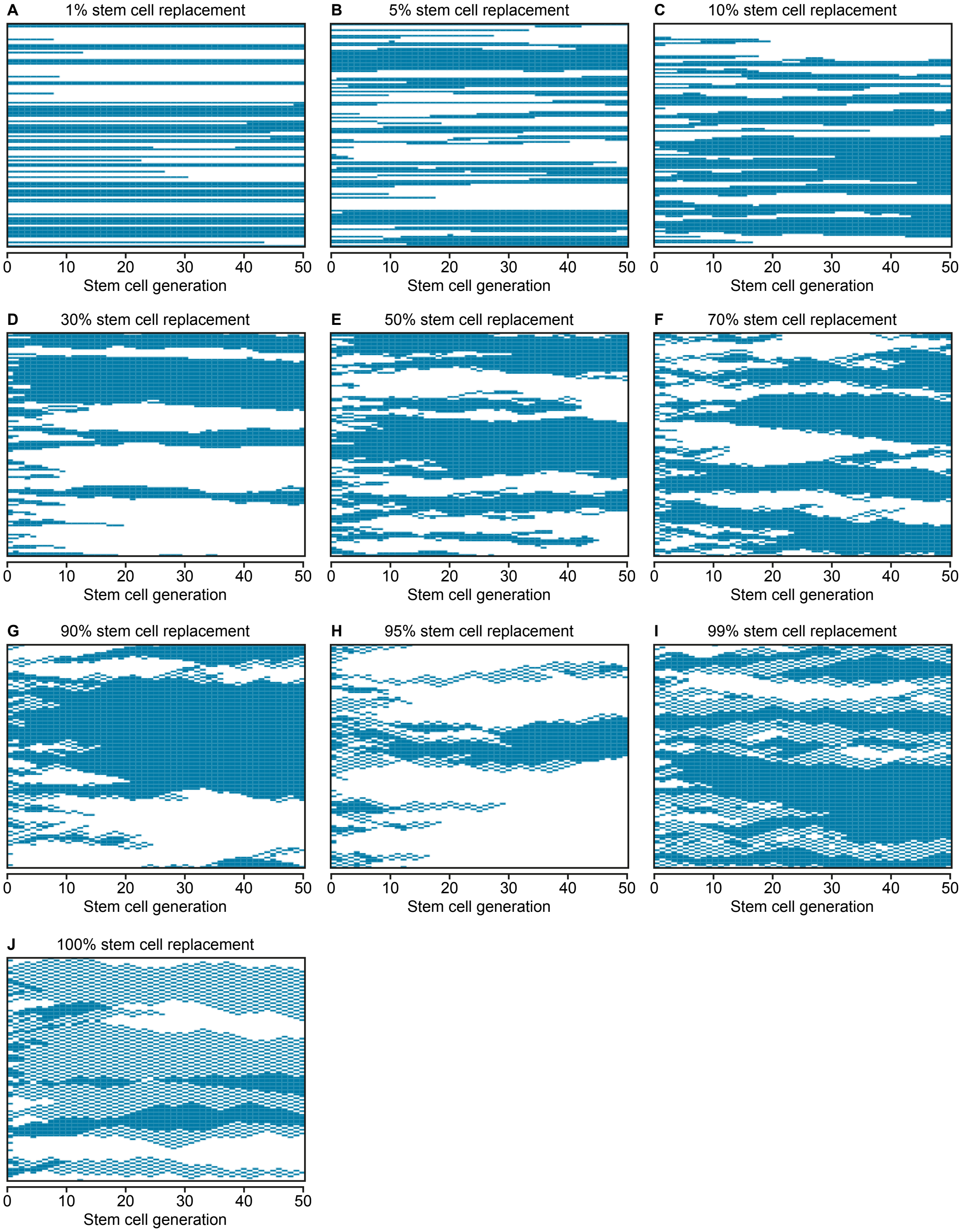

Figure S15. Example output displays, illustrating the effect of different probabilities of limbal epithelial stem cell replacement for LESC generations 0-50 in arrays with 120 LESCs, randomly distributed on the vertical axis (50% blue LESCs at G0)

(A-E) When the probability of LESC replacement was ≤50%, vertical patches of the same LESC population (blue or white) became larger at later LESC generations producing a coarser-grained vertical pattern, which would produce broader corneal stripes. **(F-J)** When the probability of LESC replacement was higher than 50%, fine-grained, vertical patterns of alternating blue and white LESCs often arose, which frequently switched back and forth at each generation. This resulted in a combination of a coarse-grained pattern, of large blue and white vertical patches of LESCs, and a fine-grained pattern of alternating individual blue and white LESCs.

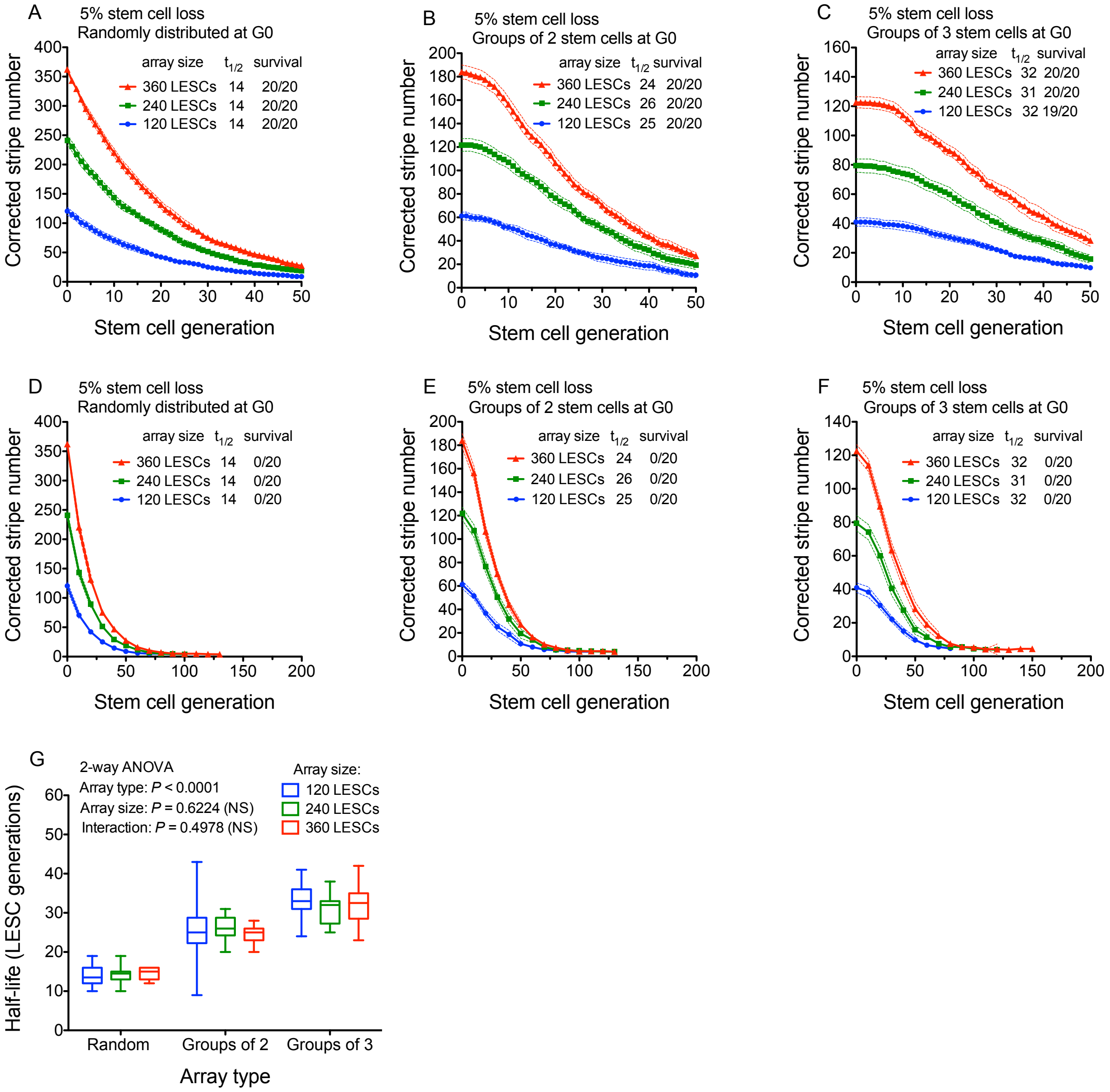

Figure S16. Effects of array size on corrected stripe number in simulations of 5% limbal epithelial stem cell loss in arrays with 50% blue LESCs at G0

(A-F) Decline in corrected stripe number (mean ± 95% CI; 20 simulations per set) over 50 (A-C) or 200 (D-F) LESC generations for arrays with 120, 240 and 360 LESCs in simulations of random distributions of blue and white LESCs (A, D), clumped arrays with LESCs arranged in groups of two at G0 (B, E) and clumped arrays with LESCs arranged in groups of three at G0 (C, F). Simulations were run for 500 LESC generations and, in (D-F), data were plotted for every tenth generation. In (A-F), the corrected stripe number half-life, shown as $t_{1/2}$ LESC generations, was determined for the mean corrected stripe number and "survival" indicates the frequency of simulations, in the set of 20, where both LESC populations survived for 50 (A-C) or 200 (D-F) LESC generations. **(G)** Box and whisker plots of the corrected stripe number half-lives, determined separately for each simulation [$t_{1/2}$ (i)] and analysed by two-way ANOVA for nine combinations of array size and array type. *P*-values are shown; NS, not significant. Box and whisker plots show the median (horizontal line within the box), upper and lower quartiles (top and bottom of boxes) and the minimum and maximum of all the data (ends of whiskers).

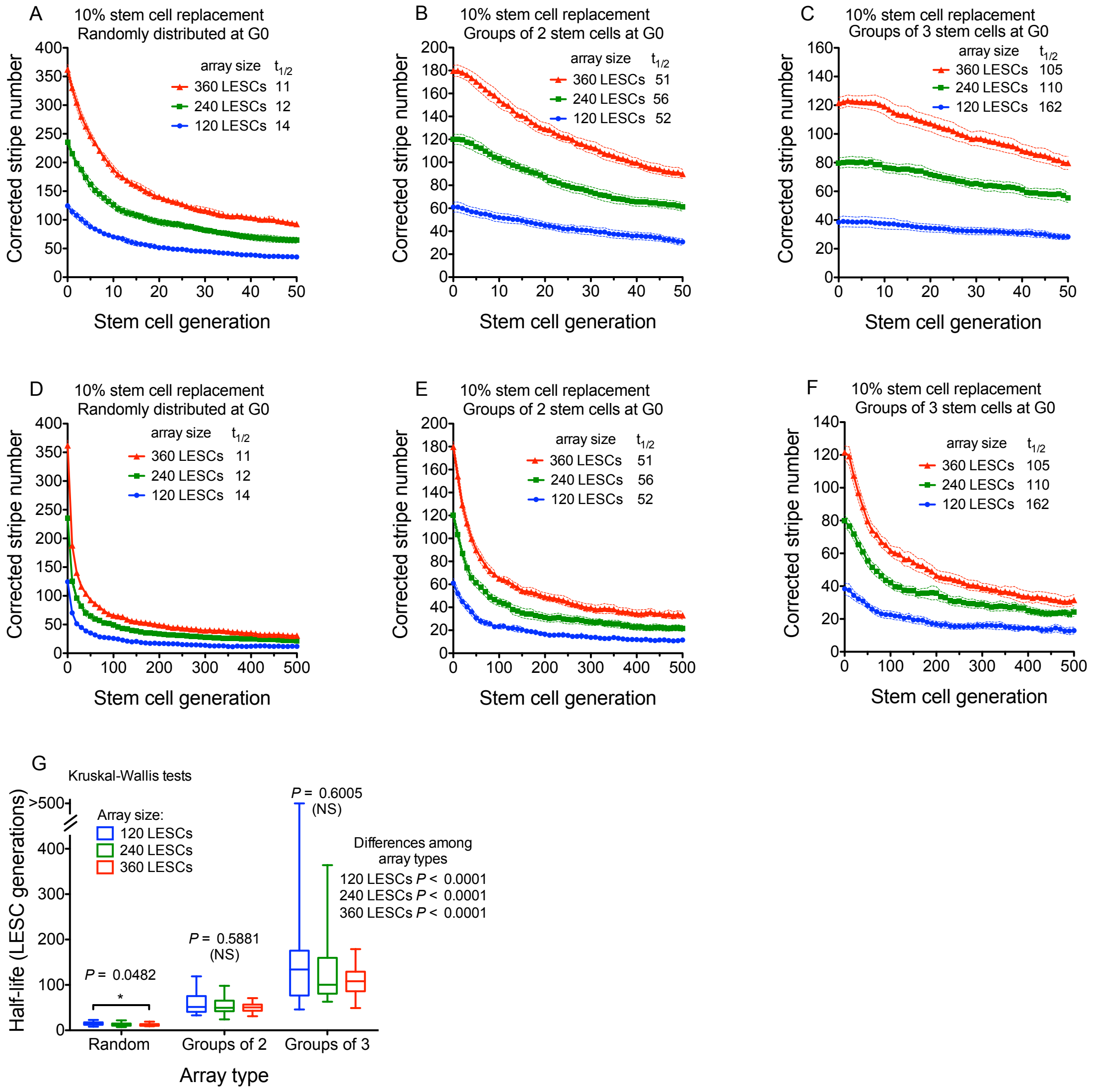

Figure S17. Effects of array size on corrected stripe number in simulations of 10% limbal epithelial stem cell replacement in arrays with 50% blue LESCs at G0

(A-F) Decline in corrected stripe number (mean ± 95% CI; 20 simulations per set) over 50 (A-C) or 500 (D-F) LESC generations for arrays with 120, 240 and 360 LESCs in simulations of random distributions of blue and white LESCs (A, D), clumped arrays with LESCs arranged in groups of two at G0 (B, E) and clumped arrays with LESCs arranged in groups of three at G0 (C, F). Simulations were run for 500 LESC generations and both LESC populations survived for 500 LESC generations in all sets of simulations. In (D-F), data were plotted for every tenth generation. In (A-F), the corrected stripe number half-life, shown as $t_{1/2}$ LESC generations, was determined for the mean corrected stripe number. **(G)** Box and whisker plots of the corrected stripe number half-lives, determined separately for each simulation [$t_{1/2}$ (i)] and analysed by non-parametric Kruskal-Wallis tests for the different combinations of array size and array type. *P*-values are shown on the graph for comparisons among array sizes and beside the graph for comparisons among array types. Dunn's multiple comparison tests were used for pairwise comparisons of array sizes, within the same array type, and the only significant difference is shown by an asterisk: **P* < 0.05; NS, not significant. *P*-values are shown; NS, not significant. The half-life was >500 for two of the 20 simulations of random arrays of 120 LESCs and the value 500 was used for the statistical tests. (Half-lives tended to be more variable in the smaller arrays and the clumped arrays, where the corrected stripe number declined more slowly.) Box and whisker plots show the median (horizontal line within the box), upper and lower quartiles (top and bottom of boxes) and the minimum and maximum of all the data (ends of whiskers).

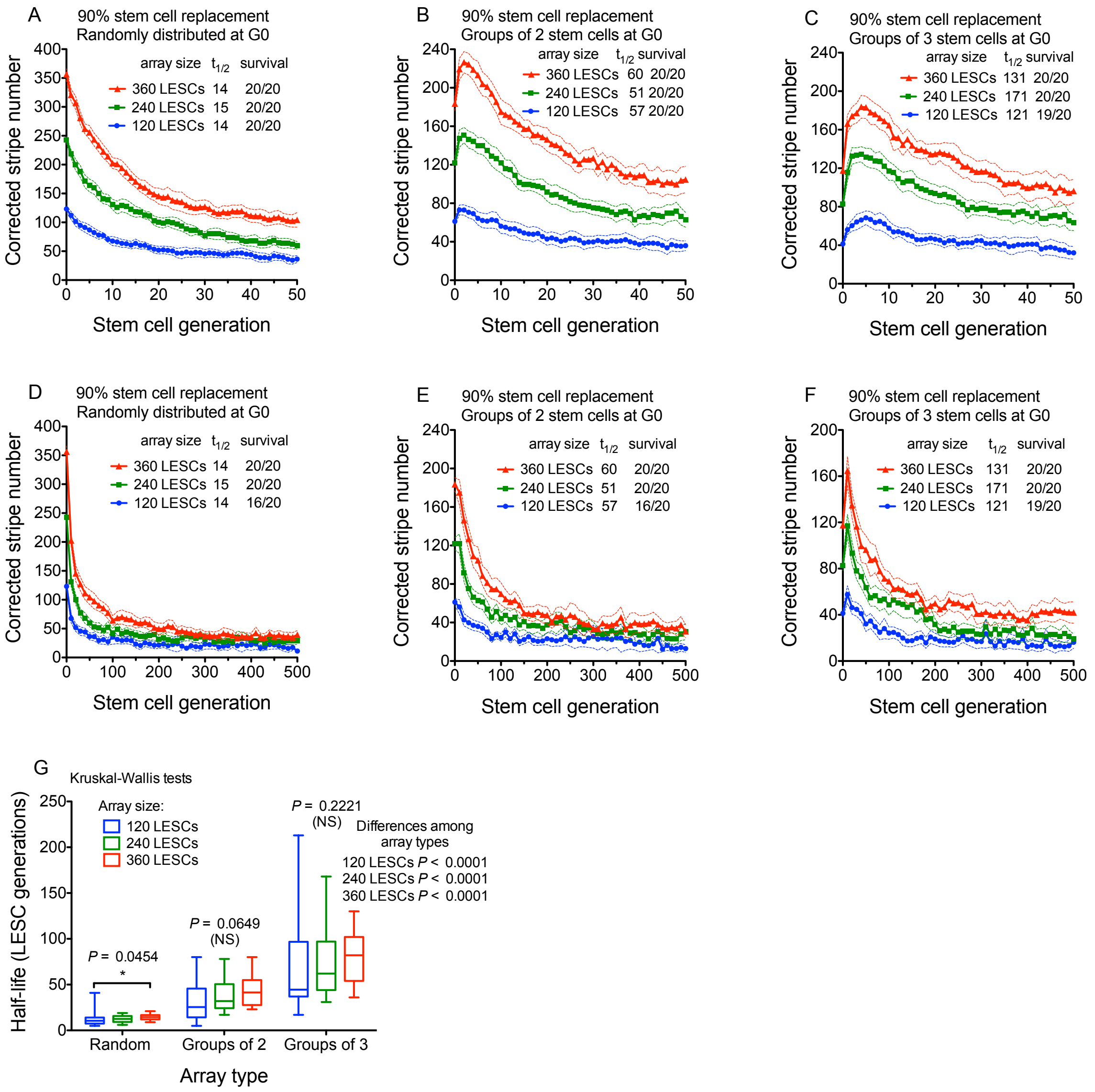

Figure S18. Effects of array size on corrected stripe number in simulations of 90% limbal epithelial stem cell replacement in arrays with 50% blue LESCs at G0

(A-F) Decline in corrected stripe number (mean ± 95% CI; 20 simulations per set) over 50 (A-C) or 500 (D-F) LESC generations for arrays with 120, 240 and 360 LESCs in simulations of random distributions of blue and white LESCs (A, D), clumped arrays with LESCs arranged in groups of two at G0 (B, E) and clumped arrays with LESCs arranged in groups of three at G0 (C, F). Simulations were run for 500 LESC generations and, in (D-F), data were plotted for every tenth generation. In (A-F), the corrected stripe number half-life, shown as $t_{1/2}$ LESC generations, was determined for the mean corrected stripe number and "survival" indicates the frequency of simulations, in the set of 20, where both LESC populations survived for 50 (A-C) or 500 (D-F) LESC generations. **(G)** Box and whisker plots of the corrected stripe number half-lives, determined separately for each simulation [$t_{1/2}$ (i)] and analysed by non-parametric Kruskal-Wallis tests for the different combinations of array size and array type. *P*-values are shown on the graph for comparisons among array sizes and beside the graph for comparisons among array types. Dunn's multiple comparison tests were used for pairwise comparisons of array sizes, within the same array type, and the only significant difference is shown by an asterisk: *$P < 0.05$; NS, not significant. (Half-lives tended to be more variable in the smaller arrays and the clumped arrays, where the corrected stripe number declined more slowly.) Box and whisker plots show the median (horizontal line within the box), upper and lower quartiles (top and bottom of boxes) and the minimum and maximum of all the data (ends of whiskers).

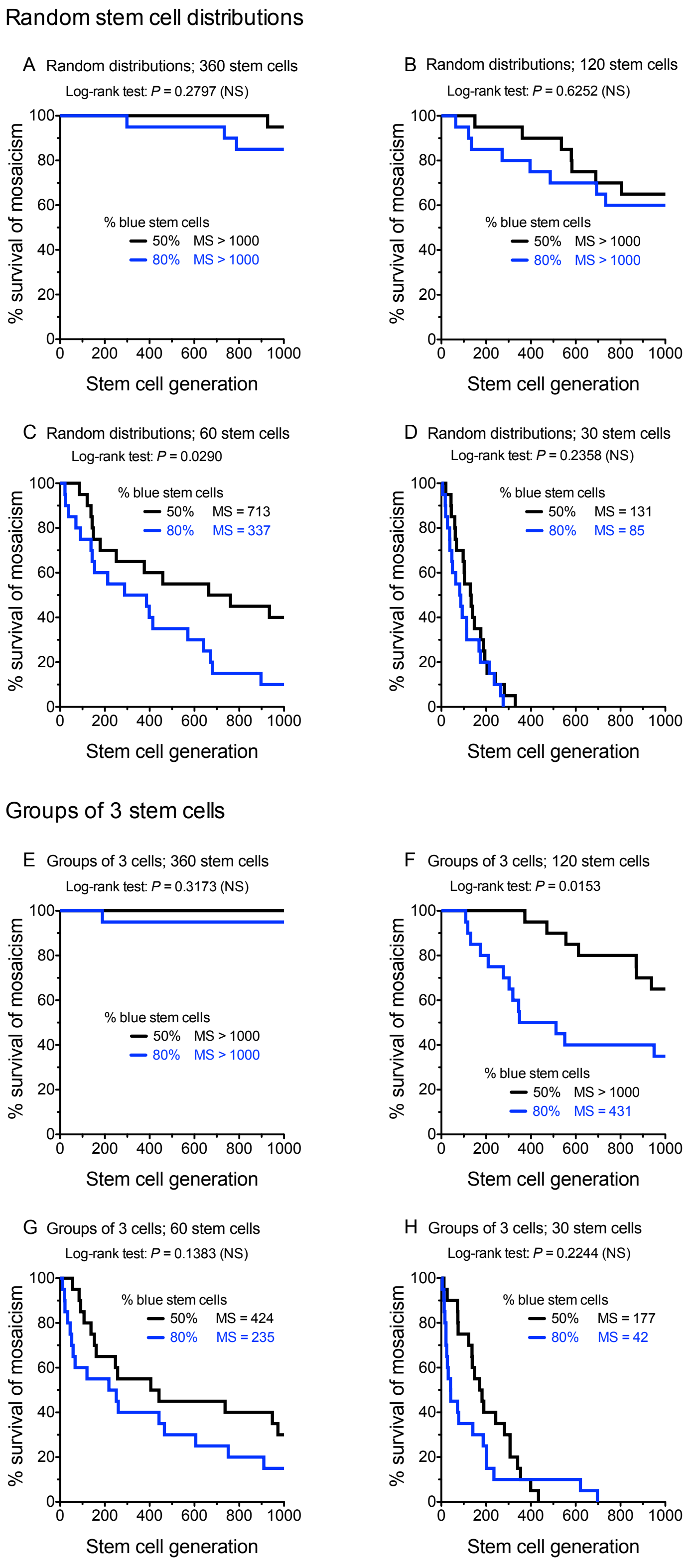

Figure S19. Comparison of loss of mosaicism in simulations of 50% limbal epithelial stem cell replacement in arrays with 50% versus 80% blue LESCs

Survival plots showing survival of mosaicism in 16 sets of 20 simulations with a 50% probability of LESC replacement per generation over 1000 LESC generations for different sizes of random arrays (A-D) and clumped arrays with groups of 3 LESCs (E-H). Differences between arrays with 50% and 80% blue LESCs were evaluated using the log-rank (Mantel-Cox) test and *P*-values are shown. Abbreviation: MS, median survival of mosaicism (in LESC generations); NS, not significant.

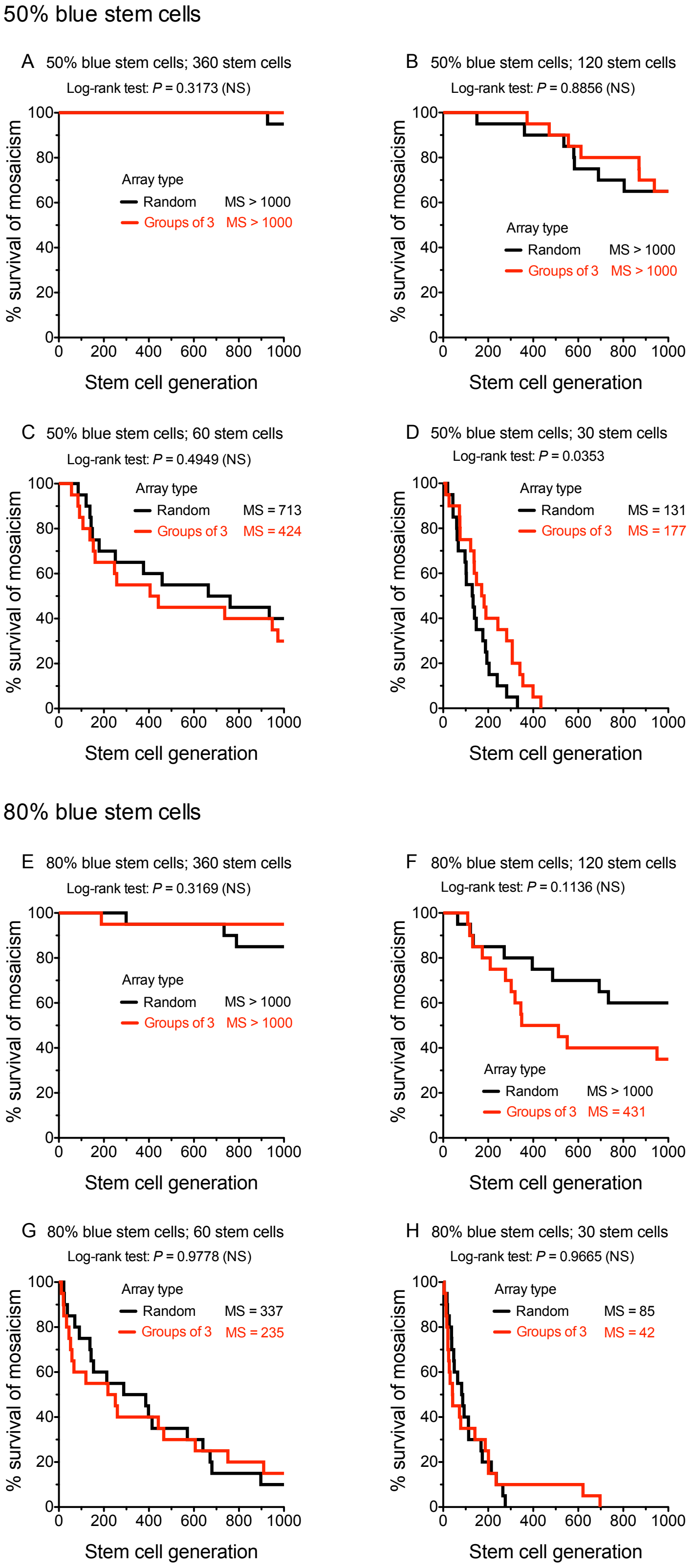

Figure S20. Comparison of loss of mosaicism in simulations of 50% limbal epithelial stem cell replacement in arrays with random LESC distributions versus groups of 3 LESCs
Survival plots showing survival of mosaicism in 16 sets of 20 simulations with a 50% probability of LESC replacement per generation over 1000 LESC generations for different sizes of arrays with 50% blue LESCs (A-D) and 80% blue LESCs (E-H). Differences between random and clumped arrays were evaluated with the log-rank (Mantel-Cox) test (*P*-values are shown). Abbreviation: MS, median survival of mosaicism (in LESC generations); NS, not significant.

SUPPLEMENTARY TABLES

Table S1. Computer simulation output

Output	Description	Calculation
**i**	Number of positive (blue) stem cells in the array	
**ii**	Number of negative (white) stem cells in the array	
**iii**	Total number of blue plus white stem cells	i + ii
**iv**	Number of blue stripes (equivalent to the number of patches of blue stem cells in a 1-dimensional circular array)* A 'patch' of blue stem cells is defined as an uninterrupted sequence of contiguous blue stem cells around the circumference	
**v**	Number of white stripes (equivalent to the number of patches of white stem cells)	
**vi**	Uncorrected stripe number	iv + v
**vii**	Proportion of blue stem cells (p)	i/iii
**viii**	Mean number of blue stem cells per blue stripe width (equivalent to the mean number of blue stem cells per blue patch length in the array)	i/iv
**ix**	Correction factor 1/(1-p)	1/(1-vii)
**x**	Corrected mean number of blue stem cells per blue stripe width (equivalent to the corrected mean number of blue stem cells per blue patch length)	viii/ix
**xi**	Corrected blue stripe number	i/x
**xii**	Corrected stripe number produced by both blue and white stem cells. This is numerically equivalent to the corrected patch number for blue plus white stem cells around the circumference. [The calculation shown is because, for closed circular arrays, the numbers of blue and white patches are equal and the corrected mean patch lengths are equal for blue and white patches.]	iii/x

* For consistency with terminology used for studies of mosaic mice, we refer to 'stripe numbers' in the text rather then 'patch numbers'

Table S2. Frequencies where both LESC populations survived for 500 LESC generations in simulations of LESC replacement in arrays with 80% blue LESCs at G0 (see Fig. S10)

Probability of LESC replacement	Limbal epithelial stem cell distribution			
	Random[††] (120 LESCs)	Groups of 2 (240 LESCs)	Groups of 3 (360 LESCs)	Groups of 4 (480 LESCs)
**10%**	19/20	19/20	20/20	20/20
**25%**	15/20	20/20	20/20	20/20
**40%[†]**	13/20	20/20	19/20	19/20

[†] Frequency of survival of both LESC populations compared among four LESC distributions for 40% LESC replacement:
2×4 Fisher's exact test: $P = 0.0025$
Chi square test for trend: $P = 0.0071$

[††] Frequency of survival of both LESC populations compared among three LESC replacement probabilities for random distributions:
2×3 Fisher's exact test: $P = 0.0579$
Chi square test for trend: $P = 0.0213$

Table S3. LESC generation (G) when one LESC population was lost before G500 in simulations of LESC replacement in arrays with 80% blue LESCs at G0 (see Fig. S10)

Probability of LESC replacement	Limbal epithelial stem cell distribution			
	Random (120 LESCs)	Groups of 2 (240 LESCs)	Groups of 3 (360 LESCs)	Groups of 4 (480 LESCs)
**10%**	G88	G459	N/A	N/A
**25%**	G49<br>G86<br>G319<br>G477<br>G497	N/A	N/A	N/A
**40%[†]**	G37<br>G48<br>G86<br>G123<br>G148<br>G307<br>G456	N/A	G192	G311

N/A, not applicable.

Table S4. Changes in LESC numbers for sets of 20 simulations of LESC loss (see Fig. S13A-D)

A. Arrays of 120 LESCs with 50% blue LESCs that were randomly distributed at G0

Probability of LESC loss (%)	LESC survival frequency		LESC number and $t_{1/2}$ (i) (Mean ± 95% CI and range)		
	G50†	G500†	G50††	G500††	$t_{1/2}$ (i) †††
**0.1%**	20/20	20/20	115±1<br>110–119	74±2<br>65–82	all >500
**0.5%**	20/20	20/20	95±2<br>89–107	10±1<br>3–17	140±8<br>113–187
**1%**	20/20	13/20	72±2<br>62–80	1±0<br>0–3	68±4<br>52–81
**5%**	20/20	0/20	9±1<br>4–16	all 0	13±1<br>10–18
**10%**	10/20	0/20	1±0<br>0–2	all 0	7±0<br>6–9
**20%**	0/20	0/20	all 0	all 0	4±0<br>3–4

B. Arrays of 360 LESCs with 50% blue LESCs in groups of 3 (clumped arrays) at G0

Probability of LESC loss (%)	LESC survival frequency		LESC number and $t_{1/2}$ (i) (Mean ± 95% CI and range)		
	G50†	G500†	G50††	G500††	$t_{1/2}$ (i) †††
**0.1%**	20/20	20/20	344±2<br>338–353	219±5<br>205–241	all >500
**0.5%**	20/20	20/20	283±4<br>265–295	29±2<br>20–45	144±6<br>120–174
**1%**	20/20	19/20	215±5<br>187–235	3±1<br>0–5	68±3<br>52–79
**5%**	20/20	0/20	27±2<br>17–36	all 0	14±0<br>12–16
**10%**	17/20	0/20	2±1<br>0–4	all 0	7±0<br>7–8
**20%**	0/20	0/20	all 0	all 0	4±0<br>3–4

† Frequency of simulations where at least some LESCs survived to generation 50 or 500 (as specified).

†† Mean number of LESCs at generation 50 or 500 ± 95% confidence interval and range.

††† $t_{1/2}$(i) is the LESC number half-life in LESC generations, defined as explained in the Materials and Methods and determined separately for each simulation. This is shown as the mean ± 95% CI and range for 20 simulations per set.

Table S5. Changes in corrected stripe numbers for sets of 20 simulations of LESC loss (see Fig. S13E-H)

A. Arrays of 120 LESCs with 50% blue LESCs that were randomly distributed at G0

Probability of LESC loss (%)	Stripe survival frequency		Corrected stripe number and $t_{1/2}$ (i) (Mean ± 95% CI and range)		
	**G50**†	**G500**†	**G50**††	**G500**††	**$t_{1/2}$ (i)**†††
**0.1%**	20/20	20/20	114±5<br>96–140	74±5<br>49–89	>500<br>443– >500[a]
**0.5%**	20/20	20/20	99±4<br>84–116	11±1<br>5–16	143±16<br>91–253
**1%**	20/20	3/20	74±4<br>56–88	4±0 (for n=3)<br>4–4[b]	68±6<br>48–103
**5%**	19/20	0/20	10±2<br>4–17	No stripes	14±1<br>10–18
**10%**	3/20	0/20	4±0 (for n=3)<br>4–4[b]	No stripes	7±1<br>5–10
**20%**	0/20	0/20	No stripes	No stripes	4±0<br>3–5

B. Arrays of 360 LESCs with 50% blue LESCs in groups of 3 (clumped arrays) at G0

Probability of LESC loss (%)	Stripe survival frequency		Corrected stripe number and $t_{1/2}$ (i) (Mean ± 95% CI and range)		
	**G50**†	**G500**†	**G50**††	**G500**††	**$t_{1/2}$ (i)**†††
**0.1%**	20/20	20/20	117±5<br>96–140	110±5<br>92–136	all >500
**0.5%**	20/20	20/20	123±5<br>96–140	27±3<br>16–40	318±19<br>245–416
**1%**	20/20	11/20	115±5<br>100–145	5±1 (for n=11)<br>4–8[b]	158±9<br>125–197
**5%**	20/20	0/20	26±3<br>16–41	No stripes	33±2<br>25–43
**10%**	7/20	0/20	5±1 (for n=7)<br>4–8[b]	No stripes	16±1<br>12–20
**20%**	0/20	0/20	No stripes	No stripes	8±0<br>6–9

† Frequency of simulations where both LESC populations survived to generation 50 or 500 (as specified), so stripes existed.

†† Mean corrected stripe number at generation 50 or 500 ± 95% confidence interval and range.

††† $t_{1/2}$(i) is the corrected stripe number half-life in LESC generations, defined as explained in the Materials and Methods and determined separately for each simulation. This is shown as the mean ± 95% CI and range for 20 simulations per set.

[a] For randomly distributed arrays with 0.1% LESC loss, the corrected stripe number half-life was >500 LESC generations in 19 simulations and 443 LESC generations in one simulation.

[b] As the corrected stripe number was not calculated when one or both LESC populations were lost, the mean 95%CI and range were calculated from less than 20 simulations (shown as n).

Table S6. Changes in corrected stripe numbers for sets of 20 simulations of LESC replacement for arrays of 120 randomly distributed LESCs with 50% blue LESCs at G0 (see Fig. S14A, B)

Probability of LESC replacement (%)	Stripe survival frequency[†]		Corrected stripe number (Mean ± 95% CI and range)		Corrected stripe number half-life[††]	
	G50	G500	G50	G500[a]	$t_{1/2}$ (i)	$t_{1/2}$
**1%**	20/20	20/20	83±4<br>72–104	33±2<br>25–45	134±20<br>87–254	115
**5%**	20/20	20/20	43±3<br>29–56	16±2<br>8–22	26±4<br>15–50	24
**10%**	20/20	20/20	30±2<br>16–39	9±1<br>4–17	12±1<br>8–18	12
**30%**	20/20	19/20	25±3<br>12–39	9±2 (for n=19)<br>4–19	5±1<br>3–9	5
**50%**	20/20	17/20	22±4<br>13–48	10±4 (for n=17)<br>4–39	4±1<br>2–8	3
**70%**	20/20	17/20	22±4<br>8–41	11±3 (for n=17)<br>4–26	7±1<br>3–18	6
**90%**	20/20	16/20	34±7<br>16–68	21±8 (for n=16)<br>8–65	12±3<br>4–34	12
**95%**	20/20	18/20	42±7<br>20–71	24±6 (for n=18)<br>4–47	19±4<br>6–37	27
**99%**	20/20	17/20	84±13<br>34–139	42±13 (for n=17)<br>4–123	62±15<br>13–129	116
**100%**	20/20	20/20	116±12<br>60–164	143±18<br>33–200	134±60 (for n=9)<br>30–273 (for n=9)[b]<br>>500 (for 11)	>500[b]

[†] Frequency of simulations where both LESC populations survived to generation 50 or 500 (so stripes existed).
[††] $t_{1/2}$ is the corrected stripe number half-life, expressed in LESC generations. It was defined as explained in the Materials and Methods and determined for the mean corrected stripe number. The $t_{1/2}$(i) value was determined separately for each individual simulation and is shown as the mean ± 95% CI and range for 20 simulations per set.

[a] As the corrected stripe number was not calculated when one or both LESC populations were lost, in some cases, the mean, 95% CI and range were calculated from less than 20 simulations (shown as n).

[b] For randomly distributed arrays with 100% LESC replacement, there was a trend for the mean corrected stripe number to increase and this mean value did not fall to half of the original value within 500 LESC generations so the $t_{1/2}$ value was recorded as >500. However, the corrected stripe number varied widely in individual simulations and $t_{1/2}$(i) values are shown for those individual simulations (9/20) where the corrected stripe number fell to half of the original value, or lower. For the remaining 11/20 individual simulations, the corrected stripe number half-life, $t_{1/2}$(i) was greater than 500 LESC generations.

Table S7. Changes in corrected stripe numbers for sets of 20 simulations of LESC replacement for clumped arrays of 360 LESCs in groups of 3 with 50% blue LESCs at G0 (see Fig. S14C, D)

Probability of LESC replacement (%)	Stripe survival frequency†		Corrected stripe number (Mean ± 95% CI and range)		Corrected stripe number half-life††	
	G50	G500	G50	G500 [a]	$t_{1/2}$ (i)	$t_{1/2}$
**1%**	20/20	20/20	118±5<br>100–152	79±4<br>68–100	471 (for n=1)[b]<br>>500 (for 19)	>500[c]
**5%**	20/20	20/20	102±4<br>88–124	41±4<br>24–57	223±29<br>116–357	222
**10%**	20/20	20/20	82±4<br>68–103	33±3<br>16–44	115±16<br>52–194	117
**30%**	20/20	20/20	64±5<br>48–84	19±2<br>12–31	52±8<br>29–91	54
**50%**	20/20	20/20	56±6<br>32–84	24±5<br>12–52	40±7<br>14–73	44
**70%**	20/20	20/20	61±6<br>36–84	23±5<br>9–46	45±6<br>33–82	48
**90%**	20/20	20/20	94±9<br>68–145	27±5<br>4–52	92±19<br>28–197	112
**95%**	20/20	20/20	132±17<br>78–216	53±7<br>29–84	162±43<br>56–474	297
**99%**	20/20	20/20	252±17<br>188–324	83±20<br>17–203	341±54 (n=15)<br>176–497(n=15) [b]<br>>500 (for 5)	>500[c]
**100%**	20/20	20/20	331±24<br>239–404	372±49<br>129–543	246 (for n=1)[b]<br>>500 (for 19)	>500[c]

† Frequency of simulations where both LESC populations survived to generation 50 or 500 (so stripes existed).

†† $t_{1/2}$ is the corrected stripe number half-life, expressed in LESC generations. It was defined as explained in the Materials and Methods and determined for the mean corrected stripe number. The $t_{1/2}$(i) value was determined separately for each individual simulation and is shown as the mean ± 95% CI and range for 20 simulations per set.

[a] As the corrected stripe number was not calculated when one or both LESC populations were lost, in some cases, the mean 95% CI and range were calculated from less than 20 simulations (shown as n).

[b] The $t_{1/2}$(i) values (± 95% CI and range, where appropriate) are shown for the individual simulations (n) where the corrected stripe number fell to half of the original value, or lower, as defined in the Materials and Methods. For the remaining individual simulations, the corrected stripe number half-life, $t_{1/2}$(i) was greater than 500 LESC generations.

[c] Where the mean corrected stripe number did not fall to half of the original value within 500 LESC generations, the $t_{1/2}$ value was recorded as >500.

References for Supplementary Material

Collinson, J.M., Morris, L., Reid, A.I., Ramaesh, T., Keighren, M.A., Flockhart, J.H., Hill, R.E., Tan, S.S., Ramaesh, K., Dhillon, B., West, J.D., 2002. Clonal analysis of patterns of growth, stem cell activity, and cell movement during the development and maintenance of the murine corneal epithelium. Dev Dyn 224, 432-440.

Mort, R.L., Douvaras, P., Morley, S.D., Dorà, N., Hill, R.E., Collinson, J.M., West, J.D., 2012. Stem cells and corneal epithelial maintenance: Insights from the mouse and other animal models, in: Kubiak, J.Z. (Ed.), Results Probl Cell Differ, 55, "Mouse Development: From Oocyte to Stem Cells". Springer-Verlag, Berlin & Heidelberg, pp. 357-394.
